# Ionic Dissolution Products of Lithium-, Strontium-, and Boron-Substituted Silicate Glasses Influence the Viability and Proliferation of Adipose Stromal Cells, Fibroblasts, Urothelial and Endothelial Cells

**DOI:** 10.1021/acsomega.4c06587

**Published:** 2024-12-04

**Authors:** Inari Lyyra, Mari Isomäki, Heini Huhtala, Minna Kellomäki, Susanna Miettinen, Jonathan Massera, Reetta Sartoneva

**Affiliations:** 1 Faculty of Medicine and Health Technology, Tampere University, Korkeakoulunkatu 3, Tampere FI-33720, Finland; 2 Faculty of Social Sciences, Tampere University, Arvo Ylpön katu 34, Tampere FI-33520, Finland; 3 Faculty of Medicine and Health Technology, Tampere University, Arvo Ylpön katu 34, Tampere FI-33520, Finland; 4 Research and Development and Innovation, Tampere University Hospital, Wellbeing Services County of Pirkanmaa, Arvo Ylpön katu 6, Tampere FI-33521, Finland; 5 Department of Obstetrics and Gynaecology, Seinäjoki Central Hospital, South Ostrobothnia Wellbeing Services County, Hanneksenrinne 7, Seinäjoki FI-60220, Finland

## Abstract

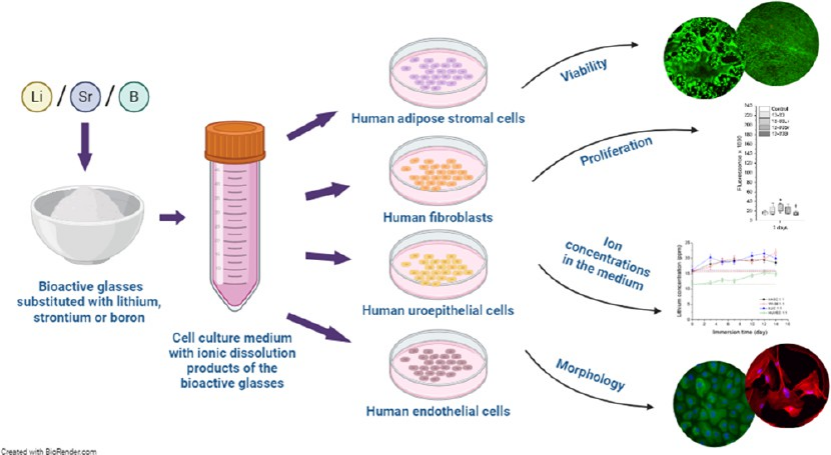

While bioactive glasses (BaGs) have been studied mainly for bone applications, studies have also shown their potential for soft tissue engineering. Incorporating therapeutic ions, such as lithium (Li^+^), strontium (Sr^2+^), and boron (B^3+^) into the BaGs, has been found to promote angiogenesis and wound healing. However, a systematic study on the impact of Li^+^, Sr^2+^, B^3+^, and the other ions in the BaGs, has not been conducted on a wide range of cells. Although the interactions between the BaGs and cells have been studied, it is difficult to compare the results between studies and conclude the impact of BaGs between cell types due to the variability of culture conditions, cells, and materials. We aim to evaluate the dissolution behavior of Li-, Sr-, and B-substituted BaGs and the effects of their ionic dissolution products on the viability, proliferation, and morphology of multiple cell types: human adipose stromal cells (hASCs), human lung fibroblasts (cell line WI-38), human urothelial cells (hUCs), and human umbilical vein endothelial cells (HUVECs). In the dissolution study, the B-substituted glasses induced a higher increase in pH and released more ions than the silicate glasses. The undiluted BaG extracts supported the viability and proliferation of all the other cell types except the hUCs. Diluting the BaG extracts to 1:10 restored the viability of hUCs but induced distinctive morphological changes. Diluting the extracts more (1:100) almost fully restored the hUC morphology. To conclude, the ionic dissolution products of Li-, Sr-, and B-substituted BaGs seem beneficial for hASCs, WI-38, hUCs, and HUVECs, but attention must be paid to the ion concentrations.

## 1 Introduction

Bioactive glasses (BaGs), such as 13-93 developed by Brink et al.,^1^ have been extensively studied for bone tissue engineering but have also raised interest in soft tissue engineering applications. One of the main advantages of BaGs is the easy incorporation of therapeutic ions. For example, lithium (Li) has been shown to increase bone mineral density and the proliferation of osteoblasts.^2,3^ Furthermore, Li-substituted BaGs have shown beneficial effects on angiogenesis and wound healing. Extracts from Li-substituted BaGs have been reported to promote the proliferation and angiogenesis of human umbilical vein endothelial cells (HUVECs), and scaffolds of Li-substituted BaGs have induced rapid ingrowth of blood vessels in rats.^4,5^

Strontium (Sr) stimulates bone formation and reduces bone resorption.^6^ In addition, a comprehensive review reported that Sr-containing BaGs have shown substantial biological improvements in bone-related applications in vitro and in vivo.^7^ In addition to their beneficial properties for bone, Sr-substituted BaGs have shown promise in promoting angiogenesis and wound healing. Sr-containing microspheres have been reported to induce early vascularization in rats.^8^ In another study by Jebahi et al.,^9^ Sr-substituted BaGs were used to treat dermal and muscular wounds in ovariectomized rats. The Sr substitution outperformed the non-Sr-containing control in muscle tissue regeneration and vascularization, resulting in fully epithelialized skin.

Boron (B) is shown to be an essential factor in osteogenesis and bone health.^10,11^ In addition, boron has shown positive effects on wound healing and angiogenesis.^12,13^ For example, in a recent article, borosilicate BaG nanofibers accelerated the healing of full-thickness skin wounds in rats.^12^ Another recent article by Decker et al.^13^ showed a positive impact of B-doped BaG on angiogenesis in ovo. A product made of boron-containing 13-93 BaG-based microfibers for treating skin wounds has also received market clearance in the United States by the U.S. Food and Drug Administration in 2016.^14^ A recent review on borate glasses by Ege et al.^14^ states that they are suitable for soft tissue repair because they convert to calcium phosphate (CaP) faster than silicate glasses, but their hydroxyapatite crystallization rate is slower.

However, comparing the results of different studies is difficult due to different cell types, bioactive glass compositions, culture conditions, and media, among other factors. This study aimed to assess the effects of ionic dissolution products from BaGs on cells isolated from different origins: human adipose stromal cells (hASCs), human lung fibroblasts (WI-38), human urothelial cells (hUCs), and HUVECs. The hASCs are abundantly available and easy to isolate, for example, by liposuction, and they can be used for many applications ranging from urethral to bone reconstruction.^15,16^ In addition, they are able to promote angiogenesis,^17,18^ which is essential in most tissue engineering applications. For a more direct indication of the effects of BaG dissolution products on endothelial cells, the HUVECs were also included in the study. The WI-38 was chosen to represent connective tissue, and it is an established cell line recommended per ISO 10993-5:2009^19^ for testing new materials. In addition, it is derived from nondiseased human tissue, which is considered as positive as the aim is in human applications.^20^ As BaGs have been raising interest in wound-healing applications, we wanted to see the effect of their dissolution products on uroepithelial cells, which represent epithelial cells in this study. In addition, hUCs are lining the lumen of the urinary tract, and they come in contact with a wide variation of excretions and ions secreted by the kidneys from the human body. One potential use for BaGs in urethral applications could be as filler materials combined with biodegradable polymers.

The bioactive glass compositions, extraction, and culture conditions were retained identical between cell types to improve comparability. To the best of our knowledge, this is the first study evaluating the effects of BaG ionic dissolution products on a broad range of cell types involving hUCs. We screened the effects of Li, Sr, and B substitution in 13-93 BaG on the dissolution properties of the glasses and the effects of the glass ionic dissolution products on the viability, proliferation, and morphology of the four cell types studied. The study’s objective was to directly compare the different BaGs in terms of dissolution behavior and soft tissue cytocompatibility of their dissolution products, as the study conditions were standardized between the BaGs.

## 2 Results

### 2.1 Glass Dissolution

**Figure 1:**
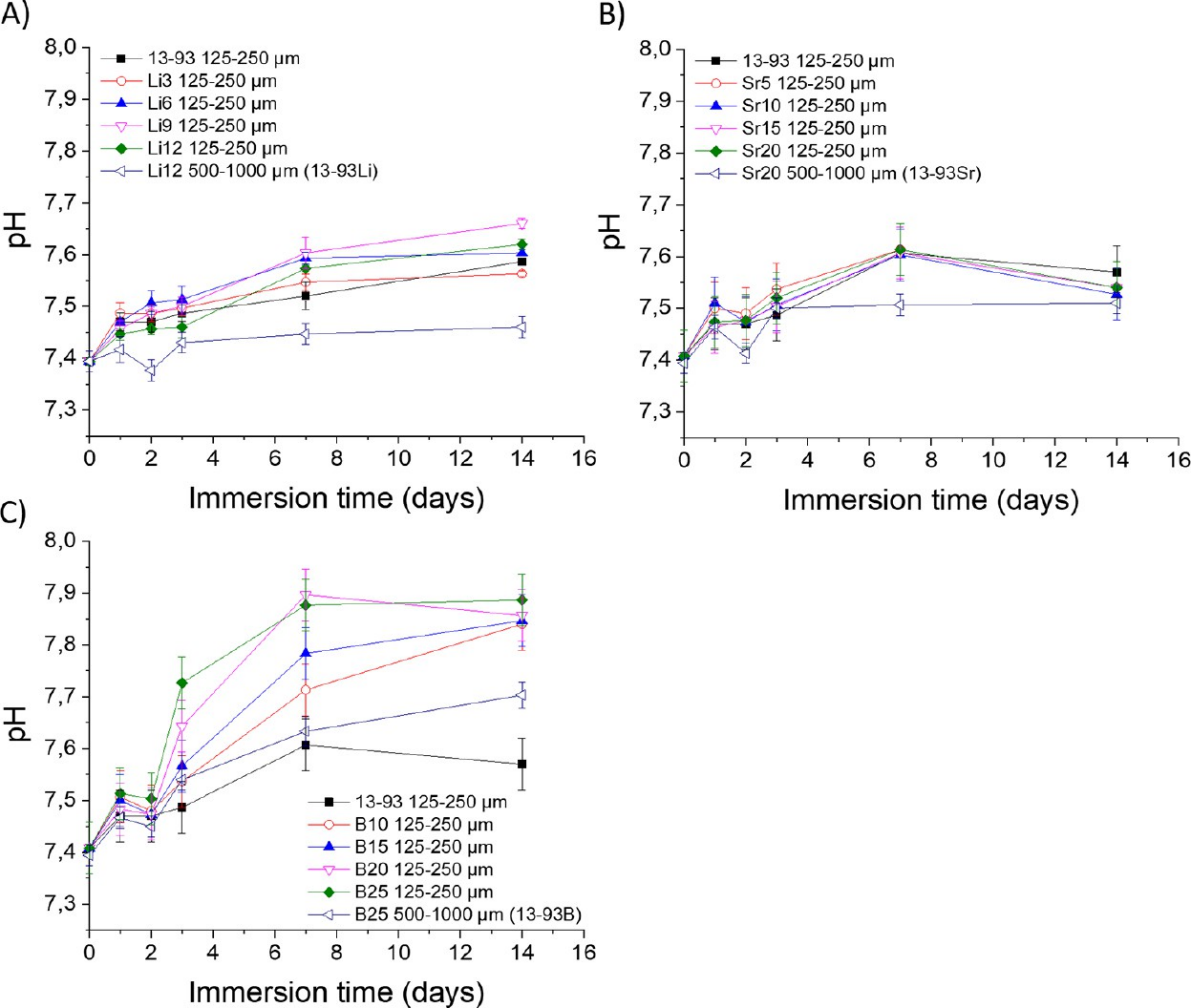
pH of SBF during the 14-day dissolution of 13-93 and (A) lithium-, (B) strontium-, and (C) boron-substituted 13-93. The pH value at the zero time point is the initial pH of the SBF before immersion of the glasses. Panels (B) and (C) have the same data for 13-93. *n* = 3. The results are presented as means with standard deviation or measurement error.

The glasses were immersed in SBF to assess their ion release and impact on the solution’s pH. The pH of the SBF increased with increasing immersion time, regardless of the BaG composition (Figure 1). While Li- and Sr-substituted glasses exhibited similar pH changes to the reference 13-93 glass, the borosilicate glasses exhibited a faster and higher pH increase than the silicate glasses. Furthermore, while increasing the B substitution to Si resulted in a higher pH, the Li substitution caused a minimal increase in pH, and the Sr substitution exhibited no significant pH changes. As expected, an increase in the particle size led to a decrease in the reaction rate; thus, the pH of the SBF upon immersion of glasses with particle sizes ranging from 500 to 1000 μm was always lower than the pH of the SBF measured upon immersion of glasses with particle sizes ranging from 125 to 250 μm.

**Figure 2:**
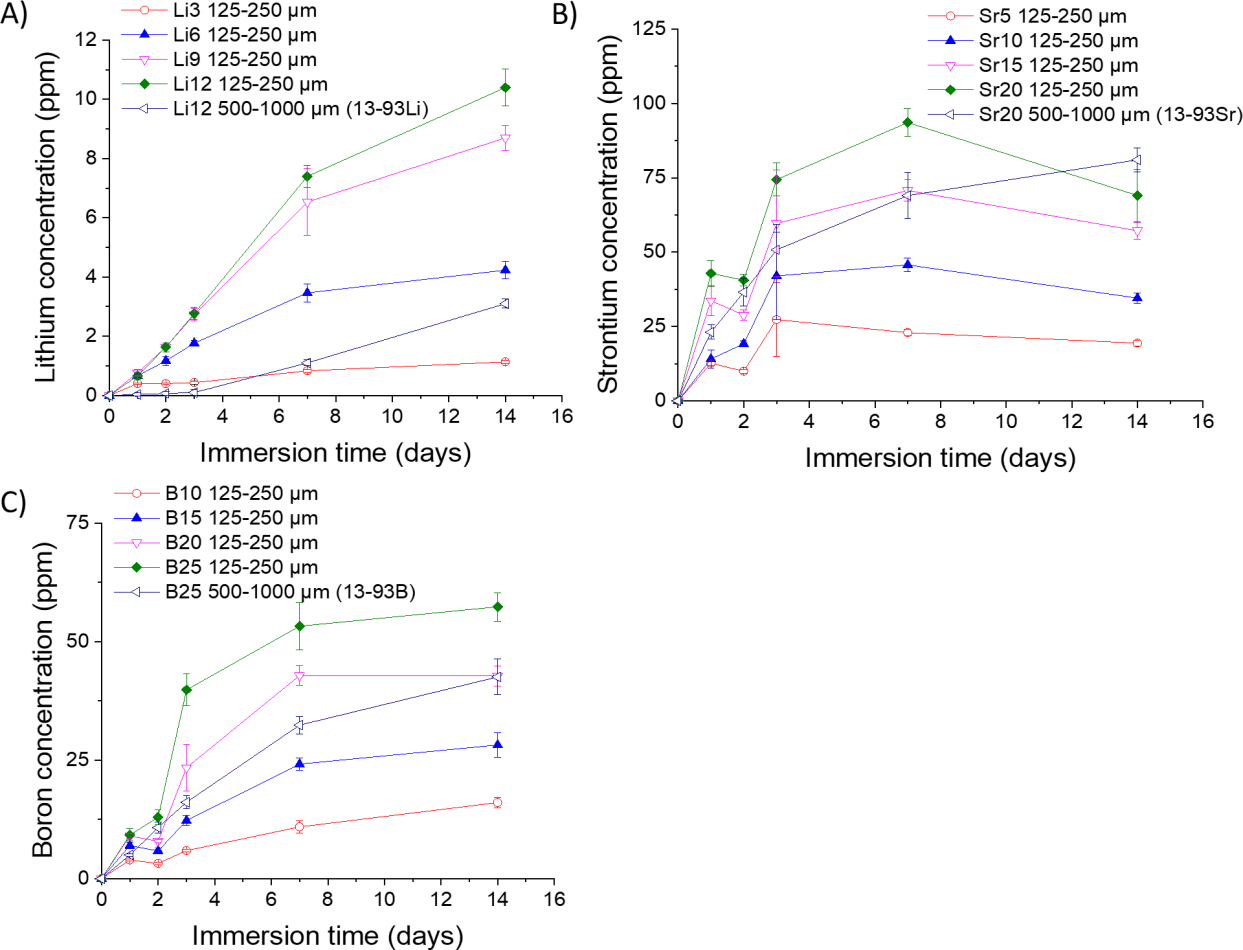
Release of (A) lithium (Li^+^), (B) strontium (Sr^2+^), and (C) boron (B^3+^) from the Li, Sr, and B-substituted 13-93 bioactive glasses during the 14-day dissolution in SBF. *n* = 3. The results are presented as means with standard deviation or measurement error.

The amount of therapeutic ions Li^+^, Sr^2+^, and B^3+^ released from the glasses increased with increasing Li, Sr, and B substitution in the glass structure, as expected (Figure 2). The 13-93Li, 13-93Sr, and 13-93B had a larger particle size, and they released less therapeutic ions than Li12, Sr20, and B25, which had the same glass composition but a smaller particle size.

The release of the other ions from the glasses, namely, Si^4+^, K^+^, P^5+^, and Ca^2+^, is presented in Figures S1–S4. The Si^4+^ and K^+^ were released similarly from all the glasses, except that the B-substituted glasses released more K^+^ than the unsubstituted 13-93 (Figures S1 and S2). In addition, the release of Si^4+^ seems to reach a plateau after 7 days of immersion (Figure S1).

The P^5+^ concentration in the solution decreases after 3 days of immersion with 13-93, Li3, and Li6, and all the B-substituted glasses (Figure S3). Furthermore, the decrease was faster with a higher B content in the glasses. In the Sr-substituted glass series, the P^5+^ concentration decreased only with the Sr5 glass, and it happened after 7 days, showing a delayed decrease of phosphate concentration (Figure S3). The decreases in P^5+^ concentration in the SBF accompanied a decrease of Ca^2+^ with the Li- and Sr-substituted glasses, and less Ca^2+^ was released with increasing Sr substitution (Figure S4). The P^5+^ decrease with the B-substituted glasses was not accompanied by a drop in Ca^2+^ concentration in the solution. In addition, the larger particle size resulted in a similar or lower release of Si^4+^, K^+^, and Ca^2+^. However, there were no decreases in P^5+^ with 13-93Li or 13-93Sr glasses compared to the corresponding glasses with a smaller particle size (Figure S3). Furthermore, the decrease in the P^5+^ concentration was more delayed with 13-93B than with B25.

### 2.2 Cell Viability and Proliferation

**Figure 3:**
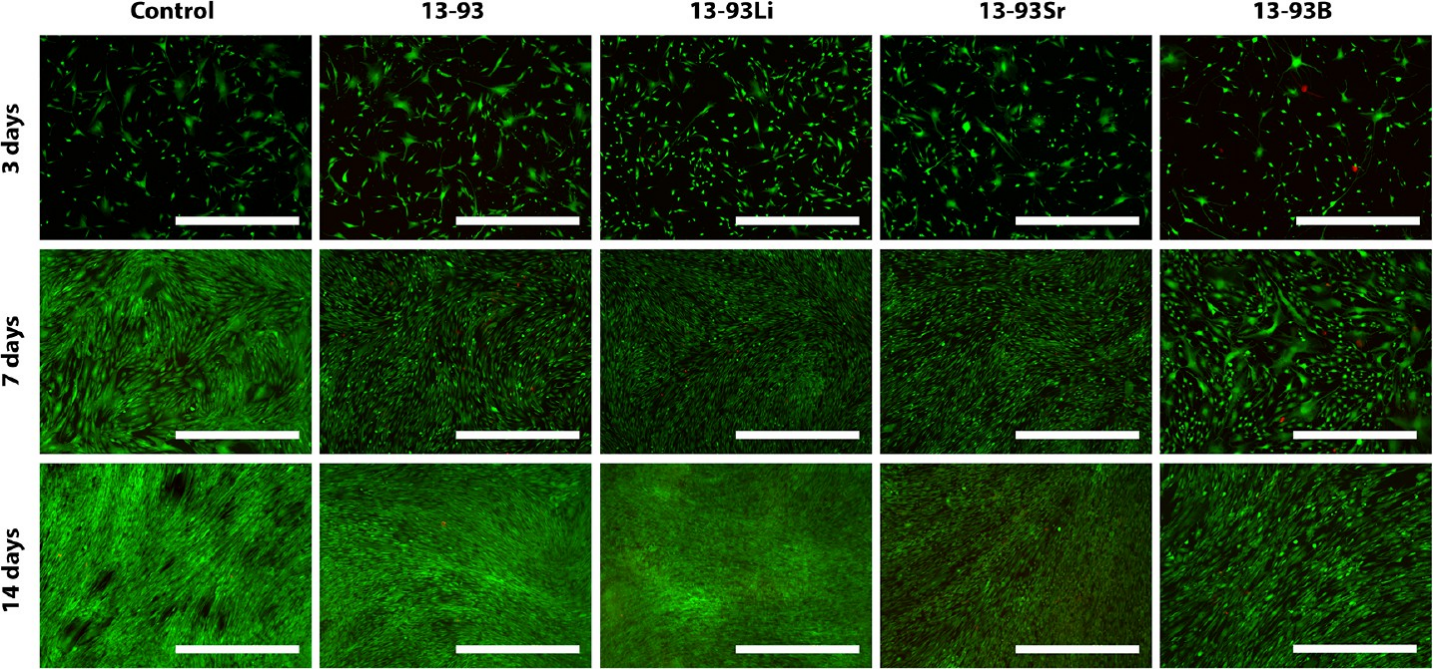
Viability of hASCs cultured for 3, 7, and 14 days in BM-based undiluted extracts of 13-93, 13-93Li, 13-93Sr, and 13-93B bioactive glasses compared to cells cultured in BM (control). The viable cells are stained green, and the dead cells are stained red. Scale bar 500 μm.

**Figure 4:**
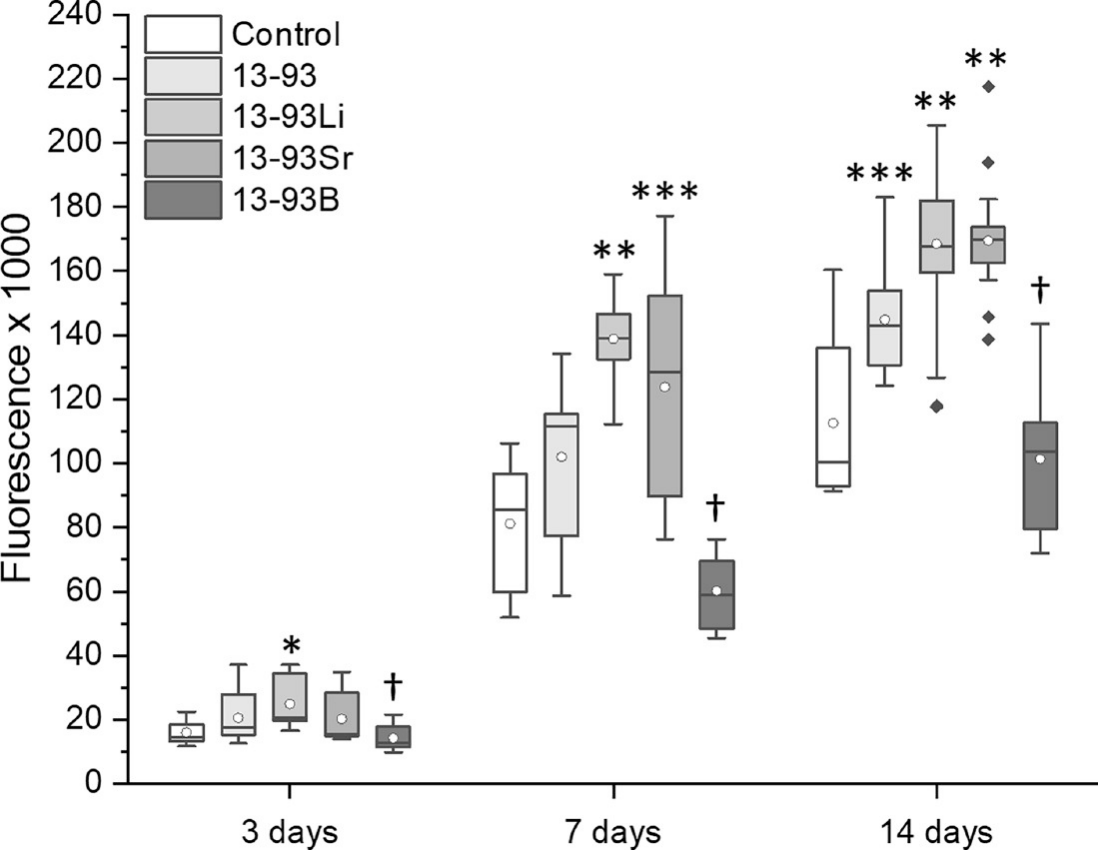
Proliferation of hASCs cultured for 3, 7, and 14 days in BM-based undiluted extracts of 13-93, 13-93Li, 13-93Sr, and 13-93B bioactive glasses compared to cells cultured in BM (control). The data are combined from three cell lines, *n* = 27. *13-93Li was significantly better than BM and 13-93Sr at 3 days; †13-93B was significantly weaker than the other extracts at all time points; **13-93Li was significantly superior to BM and 13-93 at 7 and 14 days, and 13-93Sr was significantly superior to BM and 13-93 at 14 days; ***13-93Sr was significantly superior to BM at 7 days, and 13-93 was significantly superior to BM at 14 days.

The hASCs remained viable in all extracts (Figure 3), with only a few dead cells. Based on the qualitative evaluation, the cell number increased in all the extracts within the culture period, and this was confirmed with the CyQUANT proliferation assay. However, culturing the cells in the 13-93B extract increased the cell size. Based on the quantitative analysis, there were significantly fewer hASCs in the 13-93B extract than all the other extracts at all time points (13-93 *p* < 0.001, 13-93Li *p* < 0.001, and 13-93Sr *p* = 0.01 after 3 days, *p* < 0.001 after 7 and 14 days, Figure 4). After 3 days of culture, the cell number in the 13-93Li extract was significantly higher than in BM (*p* < 0.001) and 13-93Sr extract (*p* = 0.05). After 7 and 14 days of culture, the hASC cell number in the 13-93Li extract was higher than in BM and 13-93 extract (*p* < 0.001, except *p* < 0.05 for 13-93 at 14 days). Further, at 7 and 14-day time points, the cells proliferated more in 13-93Sr extract than in BM (*p* < 0.001) and, after 14 days, more than in 13-93 extract (*p* = 0.01). In addition, at 14 days, the cells proliferated more in the 13-93 extract than in BM (*p* = 0.01).

**Figure 5:**
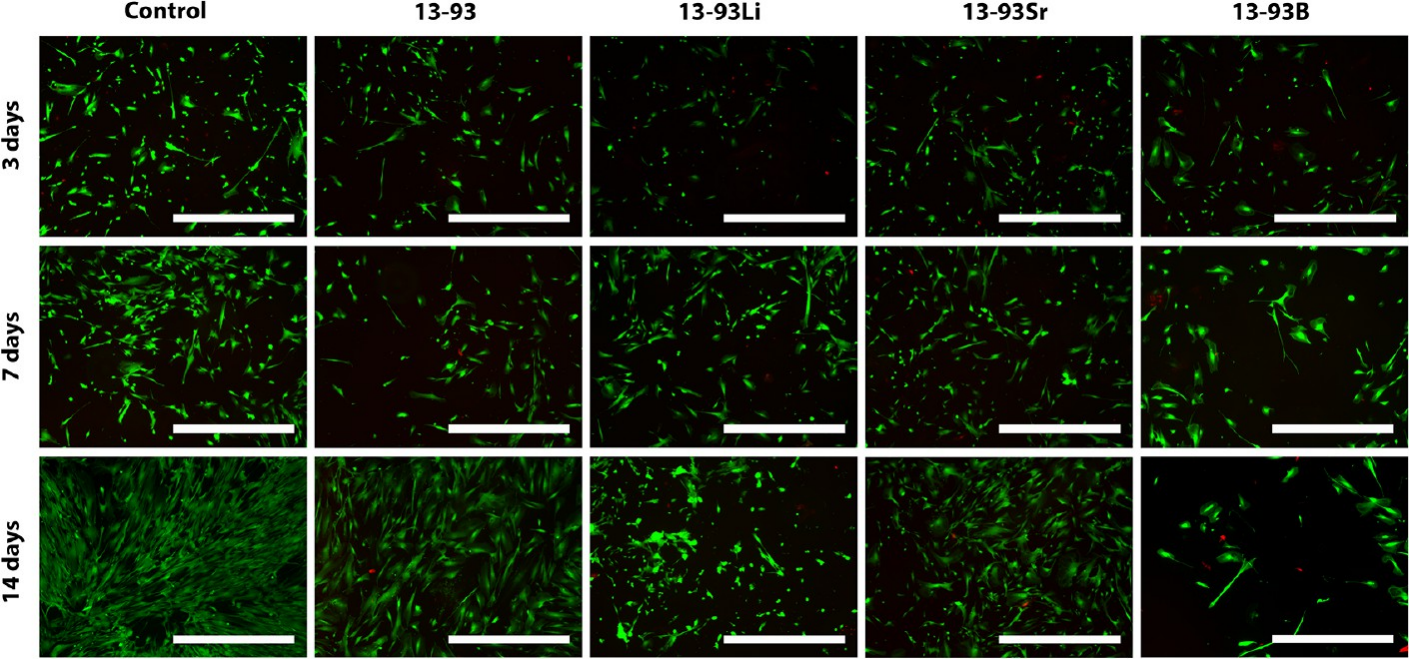
Viability of WI-38 cultured for 3, 7, and 14 days in BM-based undiluted extracts of 13-93, 13-93Li, 13-93Sr, and 13-93B bioactive glasses compared to cells cultured in BM (control). The viable cells are stained green, and the dead cells are stained red. Scale bar 500 μm.

**Figure 6:**
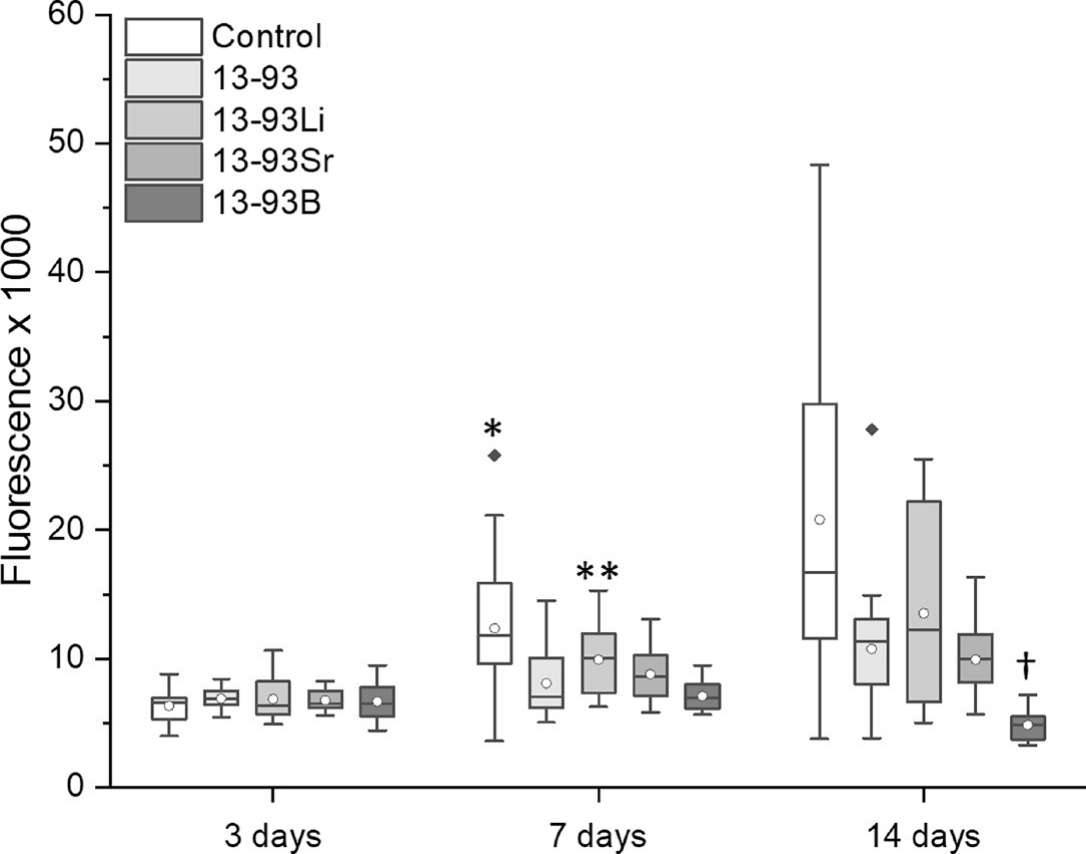
Proliferation of WI-38 cultured for 3, 7, and 14 days in BM-based undiluted extracts of 13-93, 13-93Li, 13-93Sr, and 13-93B bioactive glasses compared to cells cultured in BM (control). The data are combined from three cell lines, *n* = 27. *BM was significantly better than 13-93 and 13-93B at 7 days; **13-93Li was significantly better than 13-93B at 7 days; †13-93B was significantly weaker than BM and the other extracts at 14 days.

The extracts also supported the viability of WI-38 cells during the whole 14-day culturing period, and the number of dead cells was negligible (Figure 5). Culturing the WI-38 in the 13-93B extract also resulted in larger cells. Based on the qualitative analysis, the cell number seemed to increase during the culture period. Based on the quantitative proliferation analysis, there were no significant differences in the WI-38 cell numbers after 3 days of culture (Figure 6). After 7 days, the cells proliferated significantly more in BM than in the 13-93 and 13-93B extracts (*p* = 0.01 and *p* < 0.001, respectively) and in the 13-93Li extract more than in the 13-93B extract (*p* < 0.001). After 14 days, the cell number was significantly lower in the 13-93B extract than in BM or the other extracts (*p* < 0.001).

**Figure 7:**
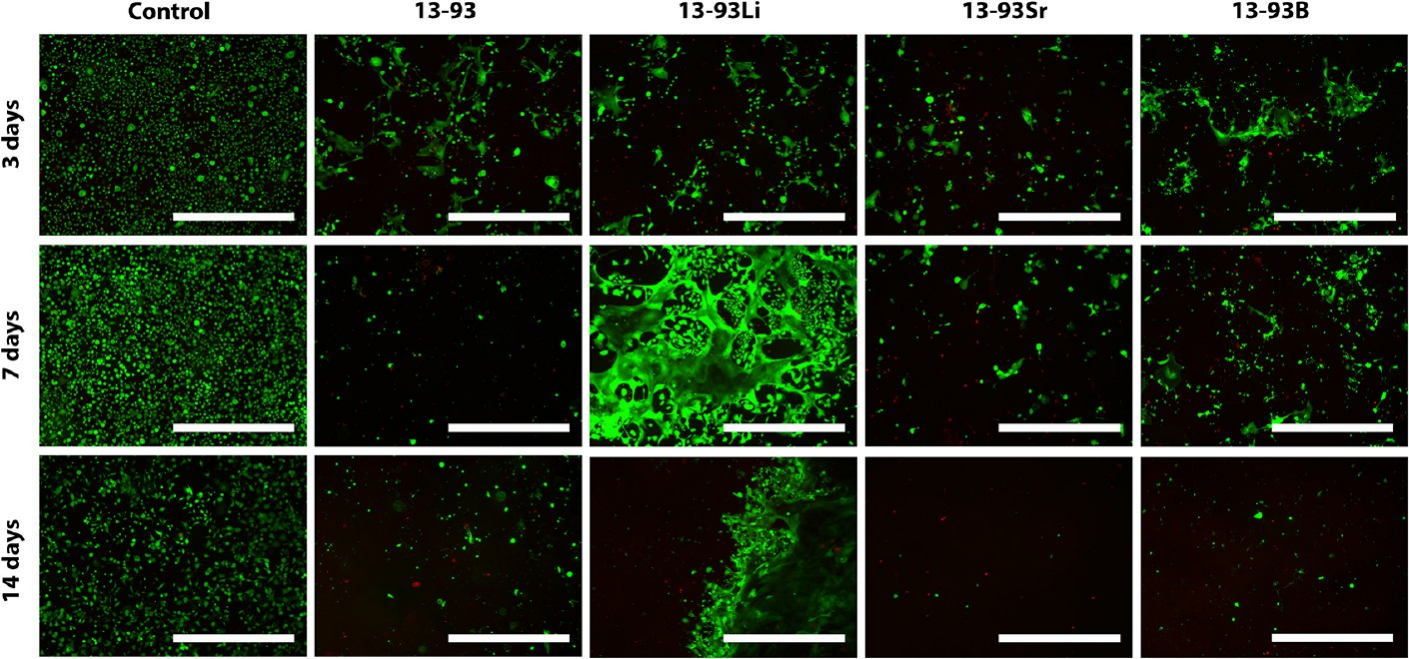
Viability of hUCs cultured for 3, 7, and 14 days in EPI-based undiluted extracts of 13-93, 13-93Li, 13-93Sr, and 13-93B bioactive glasses compared to cells cultured in EPI (control). The viable cells are stained green, and the dead cells are stained red. Scale bar 500 μm.

**Figure 8:**
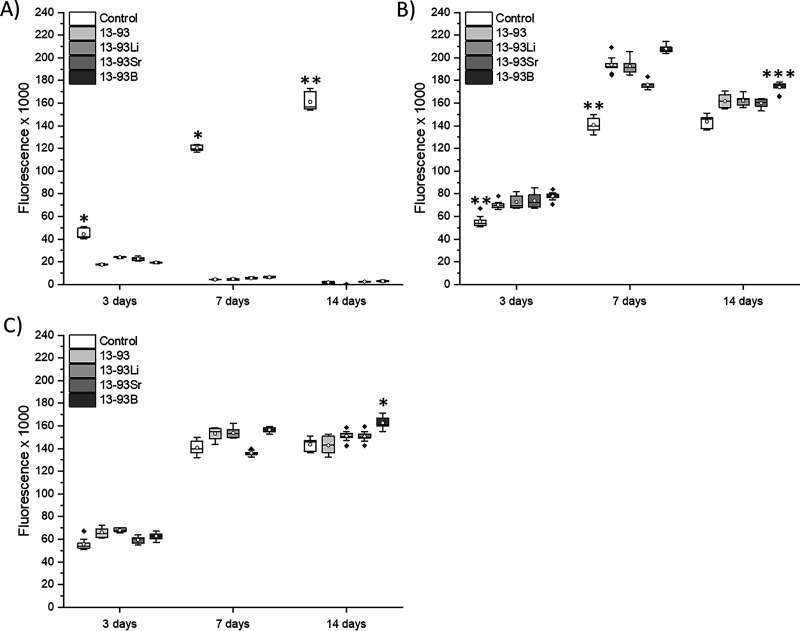
Proliferation of hUCs cultured for 3, 7, and 14 days in EPI-based (A) undiluted, (B) 1:10 diluted, and (C) 1:100 diluted extracts of 13-93, 13-93Li, 13-93Sr, and 13-93B bioactive glasses compared to cells cultured in EPI (control). *n* = 9. *Significantly higher cell number than in the 13-93 extract; **a significant difference to all the other mediums, and ***significantly better than EPI.

The undiluted extracts did not support the viability of the hUCs (Figure 7). When cultured in the undiluted extracts, at 3 days, the viability of the cells was low, and the cells adopted an abnormal morphology in 13-93, 13-93Li, and 13-93B extracts. At 7 days, the viability of the hUCs was very low in 13-93, 13-93Sr, and 13-93B extracts. In the 13-93Li extract, the cells had high viability, but they showed more morphological changes at 7 and 14 days. Based on the qualitative analysis, the cell number in all the extracts, except 13-93Li, seems to decrease during the culture. Indeed, the hUC cell number was significantly higher in the control EPI than in the undiluted 13-93 extract at all time points (*p* < 0.05, except at 14 days *p* < 0.001, Figure 8A). At 14 days, EPI supported the proliferation of hUCs over all the undiluted extracts (*p* < 0.001, except with 13-93B *p* < 0.05).

**Figure 9:**
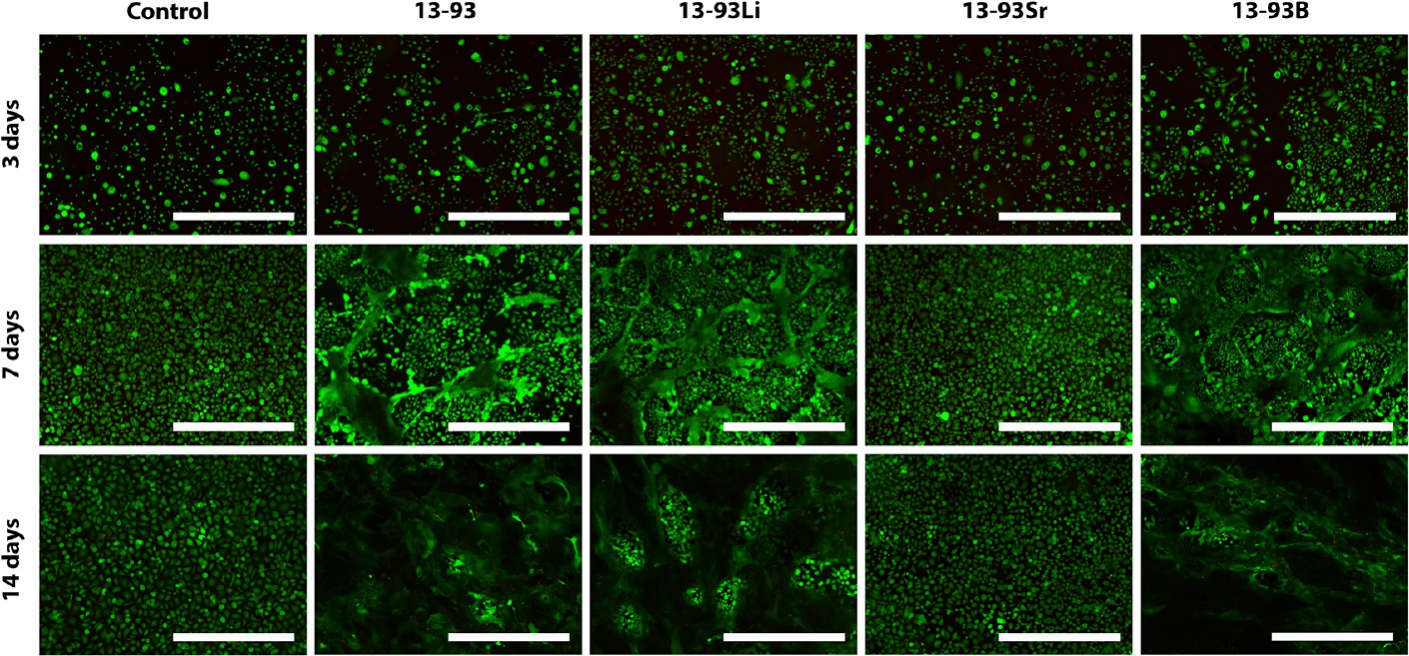
Viability of hUCs cultured for 3, 7, and 14 days in EPI-based 1:10 diluted extracts of 13-93, 13-93Li, 13-93Sr, and 13-93B bioactive glasses compared to cells cultured in EPI (control). The viable cells are stained green, and the dead cells are stained red. Scale bar 500 μm.

**Figure 10:**
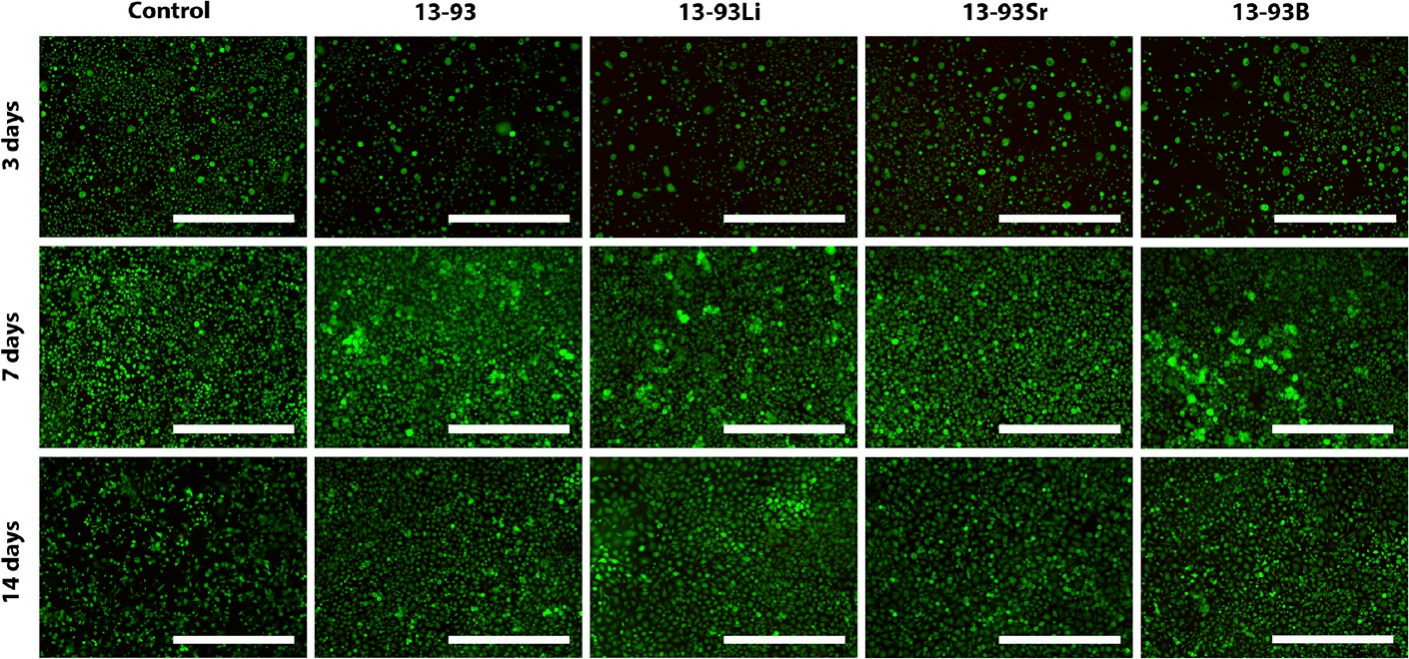
Viability of hUCs cultured for 3, 7, and 14 days in EPI-based 1:100 diluted extracts of 13-93, 13-93Li, 13-93Sr, and 13-93B bioactive glasses compared to cells cultured in EPI (control). The viable cells are stained green, and the dead cells are stained red. Scale bar 500 μm.

Diluting the extracts 1:10 increased the viability of hUCs, but similar morphology changes to those in the undiluted extracts were observed in 13-93, 13-93Li, and 13-93B extracts after 7 days of culture (Figure 9). The fraction of morphologically different cells increased at 14 days. Interestingly, 13-93Sr extract seemed to have a lesser effect on the hUCs. However, based on the L/D assay, the cell number seems to have increased as a function of culture time in all the extracts, similar to the control. Based on the proliferation assay, at 3 and 7 days, the hUC cell number was higher in the 1:10 diluted extracts than in EPI (*p* < 0.001, except *p* < 0.01 for 13-93 at 3 days and *p* < 0.05 for 13-93Sr at 7 days, Figure 8B). At 14 days, hUCs proliferated significantly better in the 13-93B extract than in EPI (*p* = 0.01), but no other differences were seen. When the extracts were diluted to 1:100, the hUCs remained viable in all the extracts (Figure 10), and only slight morphological changes were seen. At 7 days, cell clusters above the cell mat were observed in 13-93, 13-93Li, and 13-93B extracts. The cells proliferated during the culture based on the live/dead assay. The proliferation assay revealed significantly more cells in the 13-93B extract than in the 13-93 extract at 14 days (*p* < 0.05, Figure 8C).

**Figure 11:**
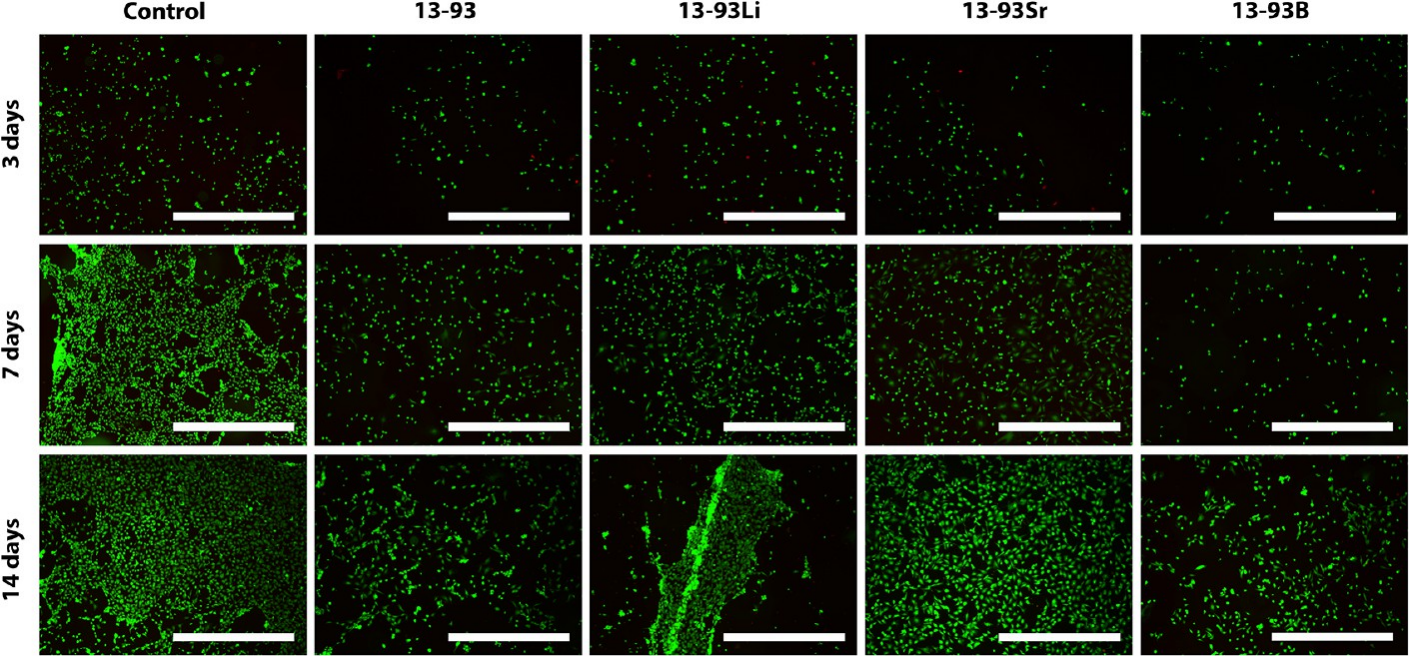
Viability of HUVECs cultured for 3, 7, and 14 days in EGM-based undiluted extracts of 13-93, 13-93Li, 13-93Sr, and 13-93B bioactive glasses compared to cells cultured in EGM (control). The viable cells are stained green, and the dead cells are stained red. Scale bar 500 μm.

**Figure 12:**
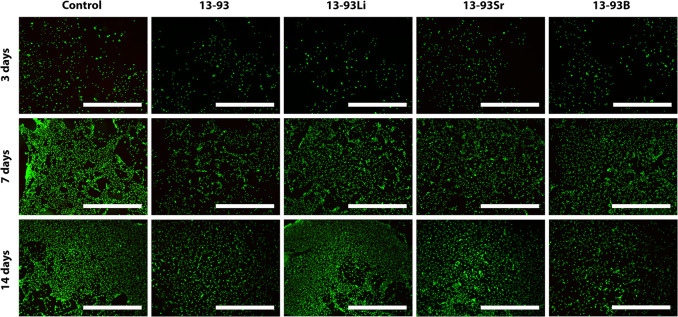
Viability of HUVECs cultured for 3, 7, and 14 days in EGM-based 1:10 diluted extracts of 13-93, 13-93Li, 13-93Sr, and 13-93B bioactive glasses compared to cells cultured in EGM (control). The viable cells are stained green, and the dead cells are stained red. Scale bar 500 μm.

**Figure 13:**
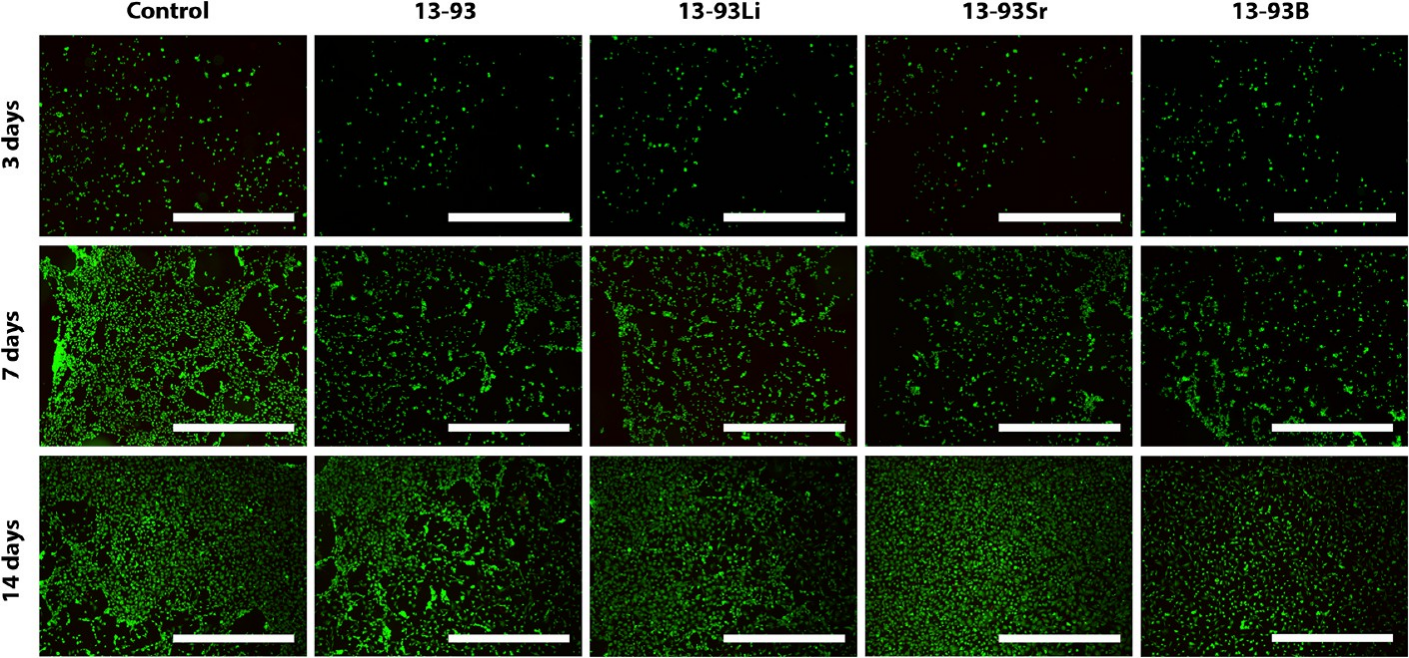
Viability of HUVECs cultured for 3, 7, and 14 days in EGM-based 1:100 diluted extracts of 13-93, 13-93Li, 13-93Sr, and 13-93B bioactive glasses compared to cells cultured in EGM (control). The viable cells are stained green, and the dead cells are stained red. Scale bar 500 μm.

**Figure 14:**
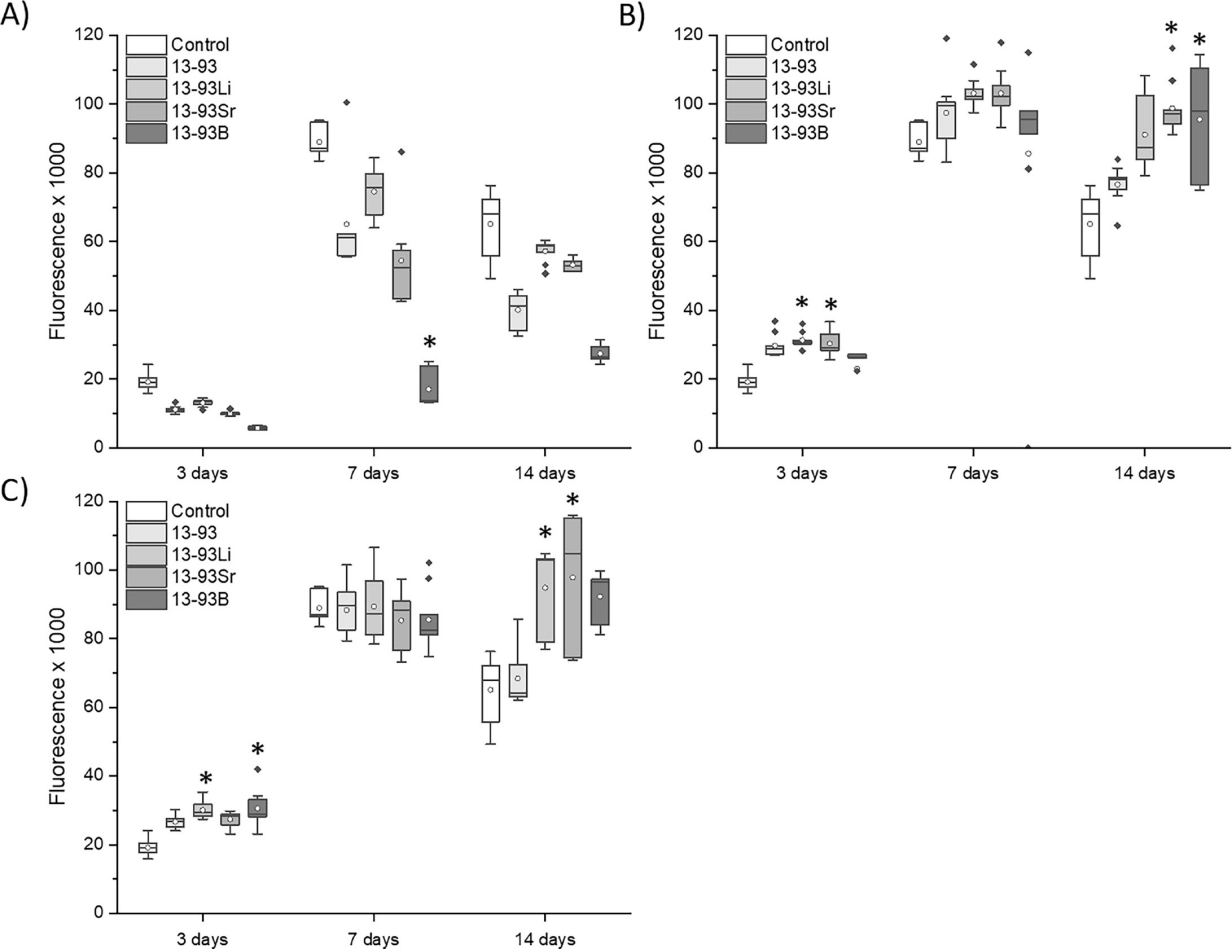
Proliferation of HUVECs cultured for 3, 7, and 14 days in EGM-based (A) undiluted, (B) 1:10 diluted, and (C) 1:100 diluted extracts of 13-93, 13-93Li, 13-93Sr, and 13-93B bioactive glasses compared to cells cultured in EGM (control). *n* = 9. *A significant difference to EGM.

HUVECs remained viable and proliferated well in all the extracts (Figures 11, 12, and 13). There were no statistically significant differences in HUVEC proliferation between the undiluted extracts and the medium or between the undiluted extracts at 3 and 14 day time points (Figure 14A). At 7 days, the cells proliferated significantly better in EGM than in the undiluted 13-93B extract (*p* = 0.01). However, diluting the extracts was beneficial for the HUVECs. When the extracts were diluted to 1:10, at a 3 day time point, the cell number was significantly higher in 13-93Li and 13-93Sr extracts than in EGM (*p* < 0.001 and *p* < 0.05, respectively, Figure 14B). At 14 days, there were significantly more cells in 13-93Sr and 13-93B extracts than in EGM (*p* < 0.05). When the extracts were diluted to 1:100, the 13-93Li and 13-93B extracts were significantly superior to EGM (*p* < 0.05) at 3 days and culturing the cells in 13-93Li and 13-93Sr extracts for 14 days yielded significantly higher cell proliferation compared to EGM (*p* < 0.05, Figure 14C).

### 2.3 Immunostaining

**Figure 15:**
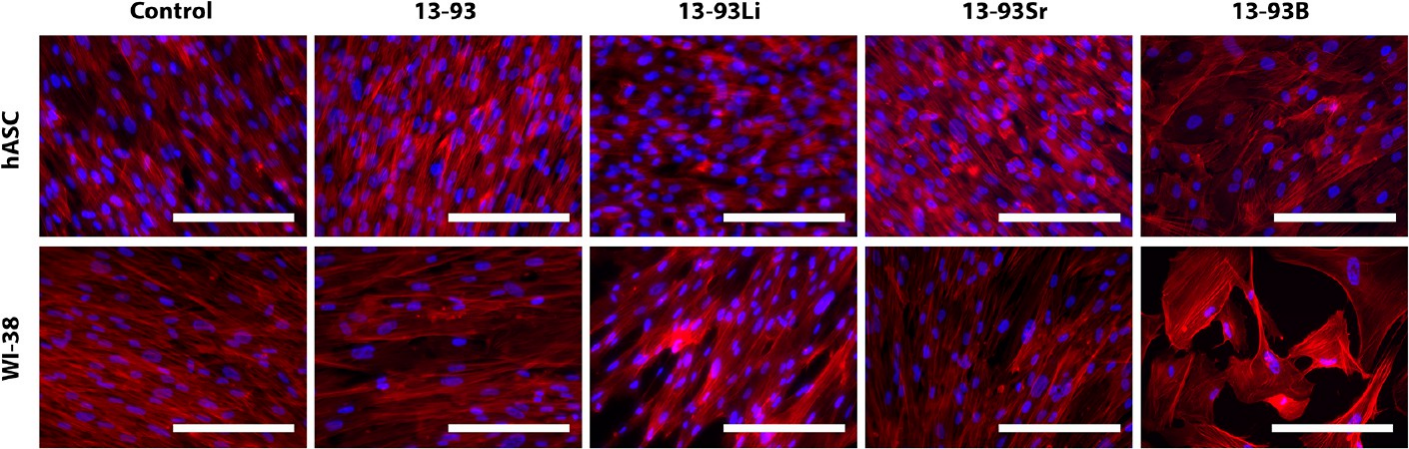
Cytoskeleton organization of hASCs and WI-38 cultured for 7 days in BM-based undiluted extracts of 13-93, 13-93Li, 13-93Sr, and 13-93B bioactive glasses compared to cells cultured in BM (control). The actin cytoskeleton was stained red with phalloidin, and the cell nuclei were stained blue with DAPI. Scale bar 200 μm.

The morphology of hASCs, WI-38, hUCs, and HUVECs was assessed via immunostaining. The actin cytoskeletons of hASCs and WI-38 were well organized and aligned when cultured in BM and undiluted 13-93, 13-93Li, and 13-93Sr extracts (Figure 15). Culturing the cells in 13-93B extract led to larger cells with a distorted morphology in both cell types; however, the effect was more prominent in WI-38.

**Figure 16:**
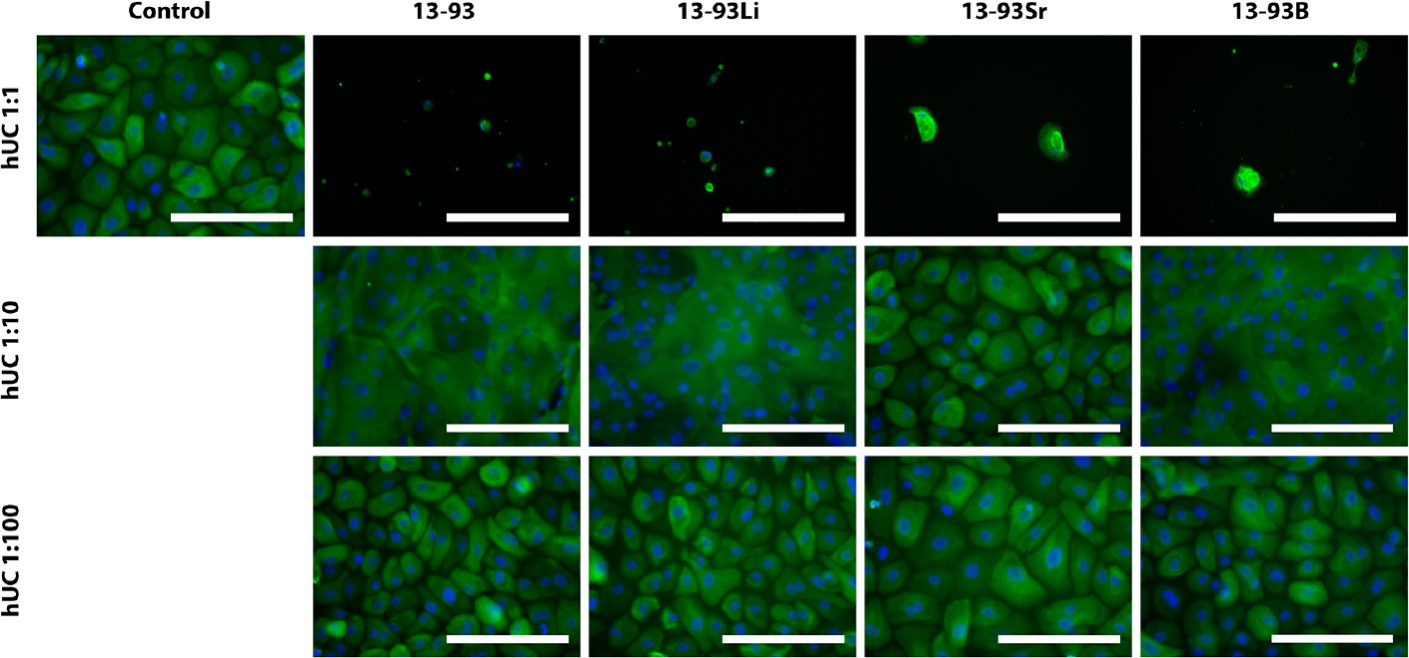
Pancytokeratin (AE1/AE3) expression of hUCs cultured for 7 days in EPI-based undiluted (1:1), 1:10 diluted, and 1:100 diluted extracts of 13-93, 13-93Li, 13-93Sr, and 13-93B bioactive glasses compared to cells cultured in EPI (control). The pancytokeratins in the cells are shown with green fluorescence, and the cell nuclei are stained blue with DAPI. Scale bar 200 μm.

The morphology and cytokeratin staining of hUCs were evaluated with pancytokeratin (AE1/AE3) staining. Culturing hUCs in the undiluted extracts resulted in round and visibly suffering cells, whereas diluting the extracts to 1:10 resulted in normal hUC morphology in the 13-93Sr extract, and significant changes in morphology were detected with 13-93, 13-93Li, and 13-93B extracts (Figure 16). The cells formed a smooth layer of large cells with undefined borders between the cells. Diluting the extracts to 1:100 normalized the hUC morphology in all the extracts. In addition, the intensity of the cytokeratin staining was similar in all of the diluted extracts.

**Figure 17:**
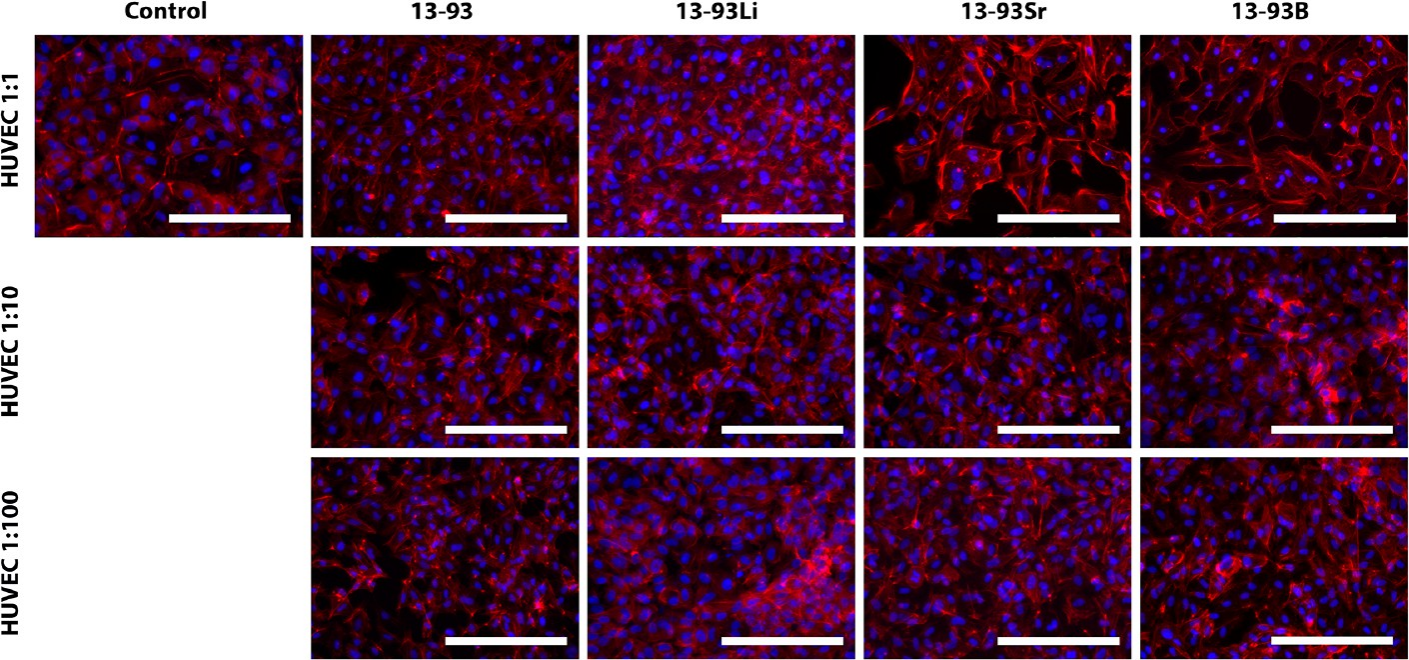
Cytoskeleton organization of HUVECs cultured for 7 days in EGM-based undiluted (1:1), 1:10 diluted, and 1:100 diluted extracts of 13-93, 13-93Li, 13-93Sr, and 13-93B bioactive glasses compared to cells cultured in EGM (control). The actin cytoskeleton was stained red with phalloidin, and the cell nuclei were stained blue with DAPI. Scale bar 200 μm.

The HUVEC morphology was normal in all of the undiluted and diluted extracts. However, the red staining was particularly strong on the edges of the cells cultured in undiluted 13-93Sr and 13-93B extracts, and the cells were larger compared to the other undiluted extracts and control (Figure 17).

### 2.4 Ion Concentrations in the Medium

**Figure 18:**
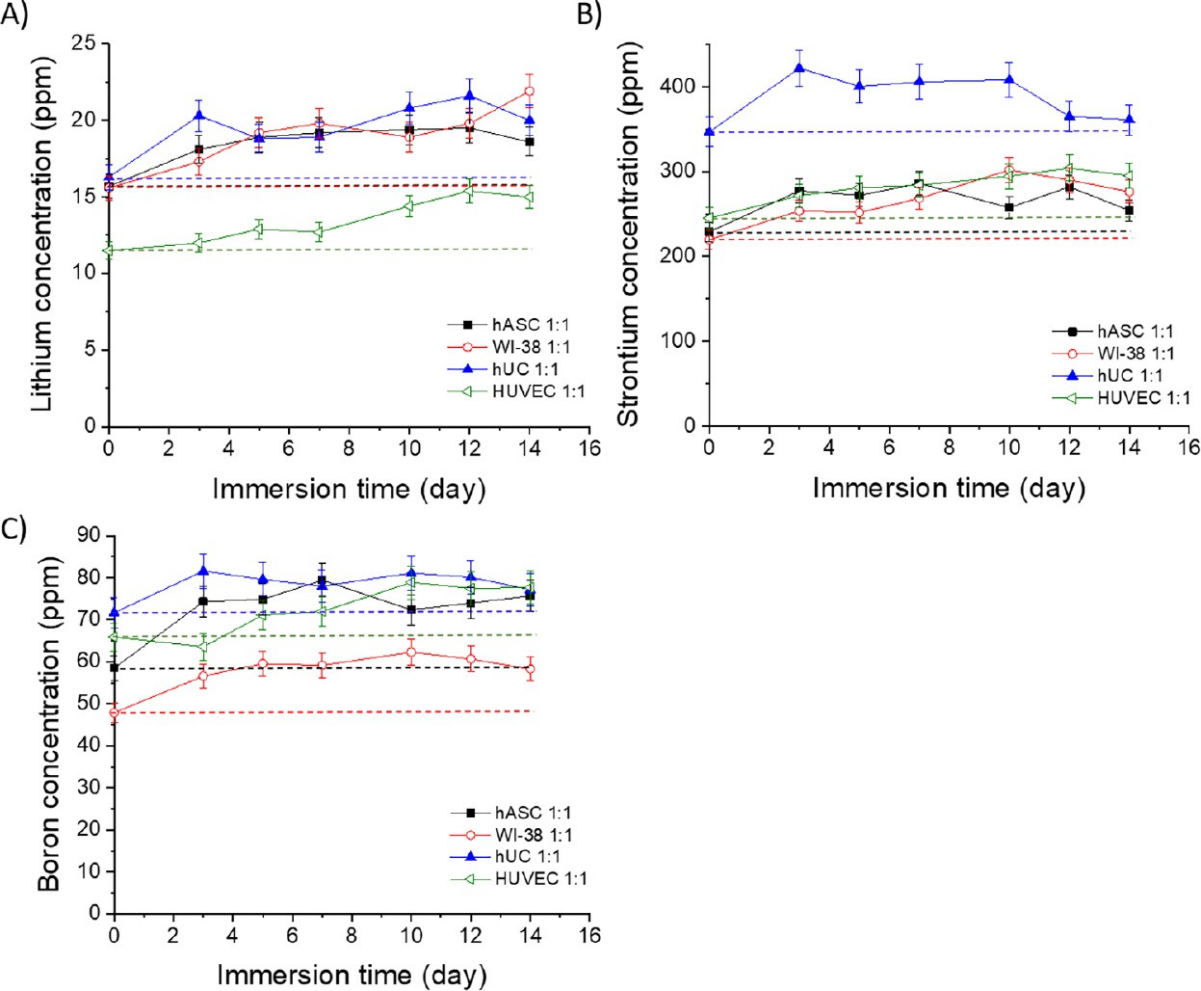
Concentrations of (A) lithium (Li^+^), (B) strontium (Sr^2+^), and (C) boron (B^3+^) in the undiluted Li-, Sr-, and B-substituted 13-93 bioactive glass extracts in the cell culture medium during the 14-day cell culture. The culture mediums were changed at 3, 5, 7, 10, 12, and 14 days. *n* = 3. The dashed lines show the initial ion concentrations in the extracts, representing the levels at a medium change. The results are presented as means with standard deviation or measurement error.

**Figure 19:**
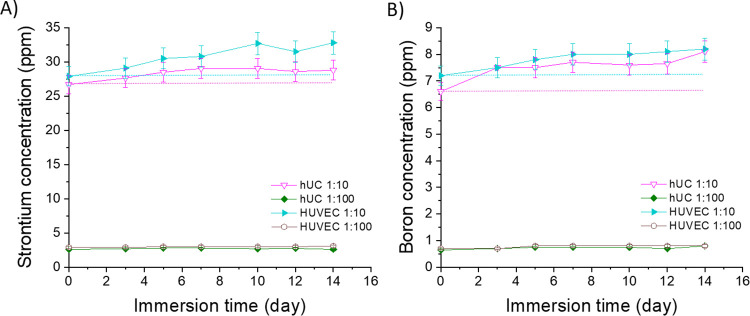
Concentrations of (A) strontium (Sr) and (B) boron (B) in the 1:10 and 1:100 diluted Sr- and B-substituted 13-93 bioactive glass extracts in the cell culture medium during the 14-day cell culture. The culture mediums were changed at 3, 5, 7, 10, 12, and 14 days. *n* = 3. The dashed lines show the initial ion concentrations of the extracts, representing the levels at medium change. The results are presented as means with standard deviation or measurement error.

The levels of the therapeutic ions Li^+^, Sr^2+^, and B^3+^ experienced by the cells during the culture in the BaG extracts are presented in Figures 18 and 19. The Li^+^ levels of the undiluted extracts increased during the culture (Figure 18A). The initial Li^+^ concentration in the BM- and EPI-based undiluted extracts was 16 ppm and increased to 19–22 ppm after 14 days of culture. The Li^+^ levels in EGM were lower than in BM, initially 12 and 15 ppm after 14 days.

The initial Sr^2+^ level in EPI was higher (347 ppm) than the initial levels of the 13-93Sr extracts made in BM or EGM (220–245 ppm, Figure 18B). The Sr^2+^ concentrations in the undiluted EPI-based extract increased to 422 ppm during the first 3 days, reached a plateau, and decreased to 361 ppm toward the end of 2 weeks. The Sr^2+^ concentrations were stable in the BM- and EGM-based extracts and were 54–295 ppm after 14 days.

The B^3+^ levels in the undiluted EPI-based extract were relatively stable at 72–82 ppm during the culture of hUCs (Figure 18C). The B^3+^ concentrations in EGM- and BM-based extracts during the culture of hASCs increased from 59–66 to 76–78 ppm during 14 days. The B^3+^ levels were the lowest in the BM-based extracts during the culture of WI-38, initially 48 and 58 ppm at 14 days.

Diluting the extracts decreased the initial ion concentrations (Figure 19). The Li^+^ concentrations of the diluted extracts are not shown because they were below the detection limit. The Sr^2+^ concentrations in the 1:10 diluted EPI- and EGM-based extracts were similar, initially 27–28 and 29–33 ppm after 14 days (Figure 19A). Diluting the extracts to 1:100 resulted in a stable Sr^2+^ concentration of 3 ppm in both EPI- and EGM-based extracts. The B^3+^ concentrations stayed at 7–8 ppm in 1:10 diluted extracts and at 0.7–0.8 in 1:100 diluted EPI- and EGM-based extracts (Figure 19B).

**Figure 20:**
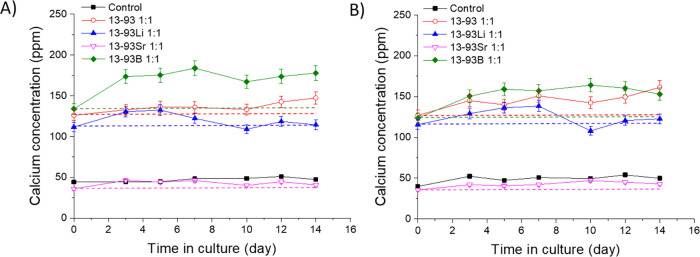
Ca^2+^concentrations of the undiluted extracts of Li-, Sr-, and B-substituted 13-93 bioactive glasses during the culture of (A) hASCs and (B) WI-38. The culture mediums were changed at 3, 5, 7, 10, 12, and 14 days. *n* = 3. The dashed lines show the initial Ca^2+^concentrations in the extracts, representing the levels at the medium change. The results are presented as means with standard deviation or measurement error.

**Figure 21:**
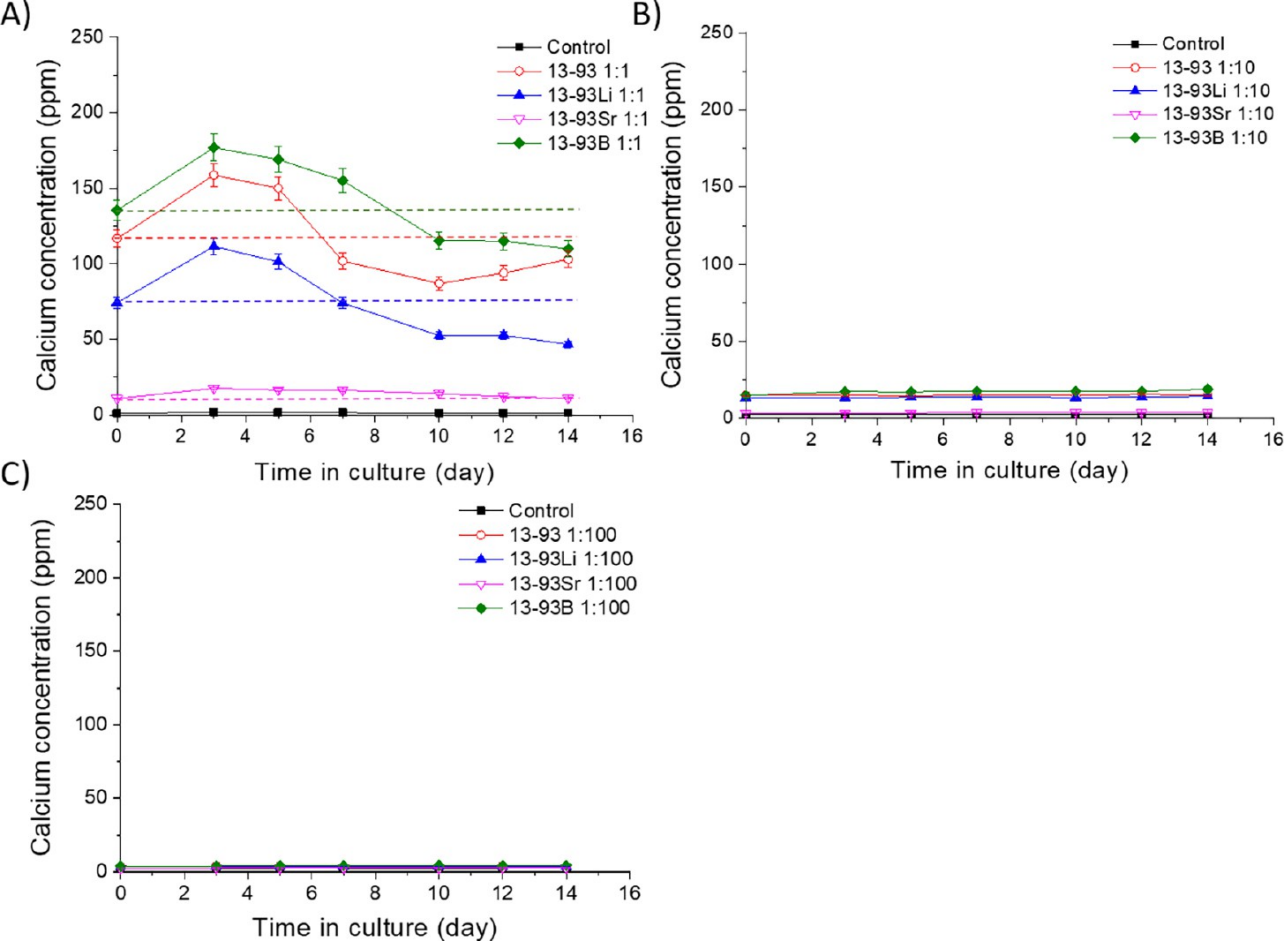
Ca^2+^concentrations of the (A) undiluted, (B) 1:10 diluted, and (C) 1:100 diluted extracts of Li-, Sr-, and B-substituted 13-93 bioactive glass in cell culture medium EPI during the 14-day cell culture of hUCs. The culture mediums were changed at 3, 5, 7, 10, 12, and 14 days. *n* = 3. The dashed lines (Panel A) show the initial Ca^2+^concentrations in the extracts, representing the levels at medium change. The results are presented as means with standard deviation or measurement error.

**Figure 22:**
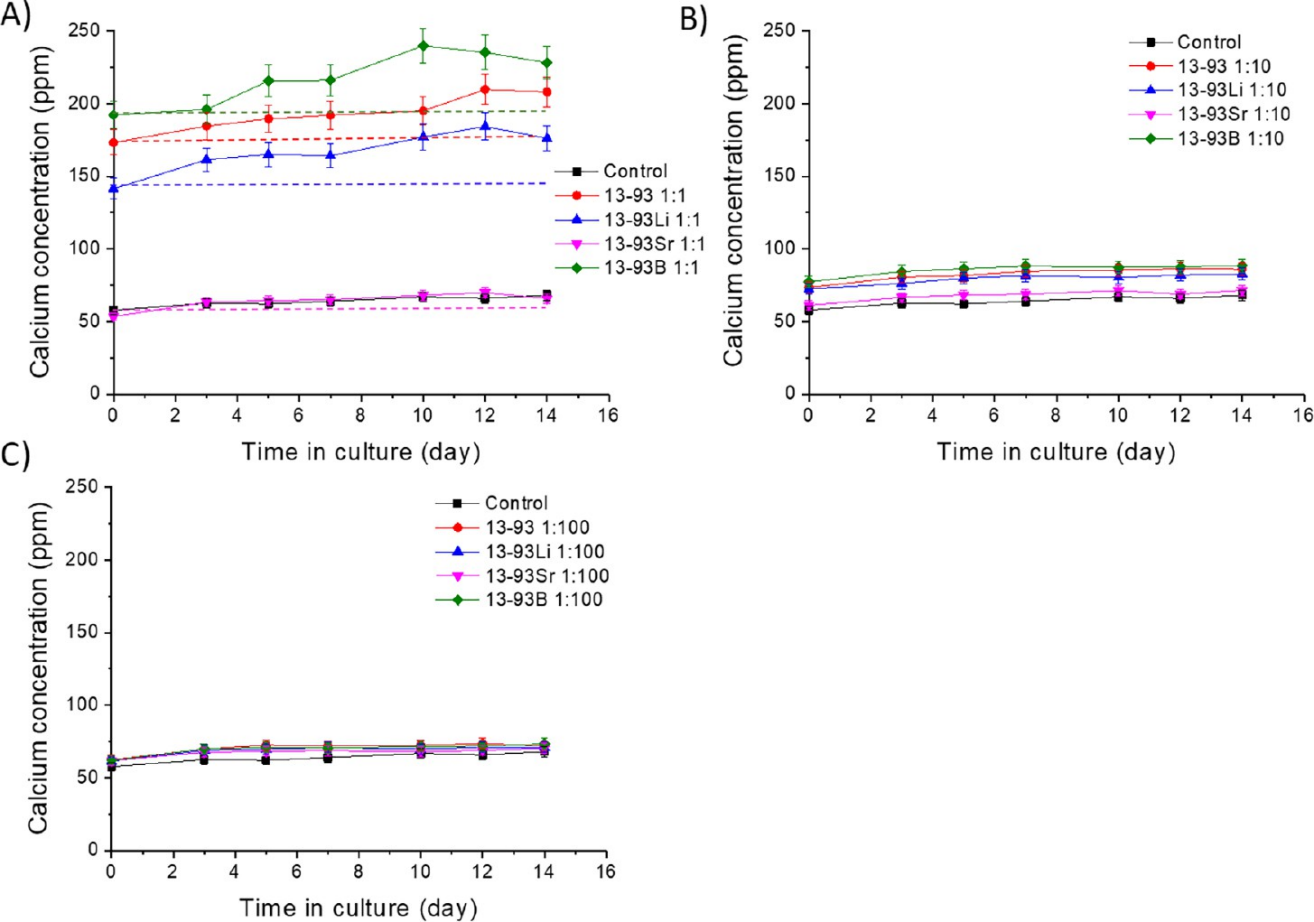
Ca^2+^concentrations of the (A) undiluted, (B) 1:10 diluted, and (C) 1:100 diluted extracts of Li-, Sr-, and B-substituted 13-93 bioactive glasses in cell culture medium EGM during the 14-day cell culture of HUVECs. The culture mediums were changed at 3, 5, 7, 10, 12, and 14 days. *n* = 3. The dashed lines (Panel A) show the initial Ca^2+^concentrations in the extracts, representing the levels at medium change. The results are presented as means with standard deviation or measurement error.

The Ca^2+^ concentrations experienced by the hASCs, WI-38, hUCs, and HUVECs during their culture in the extracts were also measured (Figures 20, 21, and 22). There was 40–54 ppm of Ca^2+^ in the control medium (BM) for hASCs and WI-38 (Figure 20). The Ca^2+^ levels during the culture of hASCs and WI-38 in the 13-93Sr extract did not distinctively differ and remained stable at 36–47 ppm. The Ca^2+^ concentrations in 13-93 and 13-93Li extracts were also relatively stable at 108–161 ppm, which was distinctively higher than those in their control medium or 13-93Sr extract (Figure 20A, B). The hASCs and WI-38 cultured in the 13-93B extract experienced the highest Ca^2+^ concentrations, 134–184 and 124–164 ppm, respectively. The Ca^2+^ levels in the 13-93B extract increased during the first 3 days and remained relatively stable for the rest of the culture period of hASCs and WI-38.

In the extracts based on EPI, the control medium for hUCs, the initial Ca^2+^ concentrations in the undiluted extracts varied greatly as a function of the glass composition (Figure 21A). The 13-93B extract contained the most Ca^2+^ (136 ppm), followed by 13-93 (117 ppm), 13-93Li (74 ppm), and 13-93Sr extracts (11 ppm). The control medium EPI contained about 1–3 ppm of Ca^2+^. The Ca^2+^ concentrations decreased from 3 days to the end of the culture period in all the other extracts except the 13-93Sr extract, reaching 47–110 ppm after the culture period. 13-93Sr extracts showed stable Ca^2+^ levels at 11–18 ppm during the culture.

During the culture, the Ca^2+^ levels in the control medium for HUVECs were 58–68 ppm (Figure 22A). Again, the Ca^2+^ concentrations of the 13-93Sr extract did not differ from the control medium. The initial Ca^2+^ levels of the other extracts were 173, 141, and 192 ppm for the 13-93, 13-93Li, and 13-93B extracts, respectively. Their Ca^2+^ concentrations increased with increasing culture time, reaching 208 (13-93), 176 (13-93Li), and 228 ppm (13-93B) after 14 days of culture with HUVECs.

As expected, diluting the extracts to 1:10 and 1:100 dilutions decreased the concentration of Ca^2+^ in the extracts (Figures 21B, C and 22B, C). With increasing dilutions, the Ca^2+^ concentrations of the extracts came closer to the ion concentration of the control mediums EPI and EGM. In the diluted extracts, the Ca^2+^ levels remained stable during the hUC culture at 4–19 ppm (1:10) and 2–4 ppm (1:100), and with HUVECs at 61–89 ppm (1:10) and 62–74 ppm (1:100).

## 3 Discussion

### 3.1 Glass Dissolution

The Li-, Sr-, and B-substituted BaGs were immersed in SBF to study their dissolution. The pH of the SBF increased during the dissolution of BaGs (Figure 1). The early stages of glass dissolution involve leaching the alkaline and alkaline-earth ions, which are then replaced in the glass with H^+^ ions, changing the pH of the solution to be more alkaline. The Li- and Sr-substituted glasses induced a similar pH increase to 13-93 (Figure 1), and similar results have been reported with Sr-substituted S53P4 and with BaGs with Li substitution, Sr substitution, and a binary Li and Sr substitution.^21,22^ Li and Sr substitute for alkali or alkaline earth metals in the glass structure. Li substitutes Na and K, whereas Sr substitutes Ca. Li and Sr behave the same in dissolution compared to the ions they substitute. Therefore, no remarkable changes from the pH changes induced by 13-93 were expected due to the Li and Sr substitution. However, Sr-substituted glasses have two properties, that, in this case, balance each other out regarding pH changes. Sr^2+^ is slightly bigger than Ca^2+^, so Sr^2+^ makes the glass network more open, accelerating the degradation. On the other hand, Sr increases the glass density as it has a higher molecular weight than Ca. The dissolution was done with a constant mass of glass particles in the solution, which led to fewer glass particles and a smaller available surface area. Increasing Sr content further decreases the surface area, hinders pH increase, and balances out the accelerating impact of the more open structure in the Sr-substituted glasses.^21^

The borosilicate glasses exhibited a faster and higher pH increase than the silicate glasses, which was expected due to the higher dissolution rate of borosilicate glasses.^23^ Both B and Si are network formers in the glass. However, B forms BO_3_ triangles and BO_4_ tetrahedra in the glass, disrupting the tetrahedra network of Si and O.^24,25^ This behavior leads to a more open structure and faster dissolution. In addition, borosilicate glasses form two phases: a silica-rich and a borate-rich phase, of which the borate phase dissolves faster. The more there is B, the more open the glass network and the higher the pH increase, as seen in our results. The larger particles exhibited a decreased dissolution rate due to the decreased surface area.

The therapeutic ions released from the glasses increased with increasing Li, Sr, and B concentrations in the glasses (Figure 2), as expected. The plateau detected in the release of Si^4+^ after 7 days (Figure S1) suggests that all the soluble Si had leached out and silica gel repolymerization had occurred, as expected from the dissolution mechanism of silicate bioactive glasses.

The decrease in P^5+^ and Ca^2+^ concentrations in the SBF, seen with 13-93, Li3, and Li6 (Figure S3), is associated with the precipitation of a reactive CaP layer. Interestingly, it seems that increased concentrations of Li^+^ together with decreased concentrations of Na^+^ and K^+^ hinder CaP formation. Thus, the bioactivity of Li9 and Li12 decreased slightly compared to the other glasses in the Li-substituted glass series. Previously, Na–Li substitution has been reported to delay the hydroxyapatite formation at lower Li substitutions (3 and 7 wt %) in 45S5 glass compared to unsubstituted 45S5 and 12 wt % Li-substituted 45S5.^26^ The fact that the delayed CaP precipitation is seen at higher Li substitutions compared to those in the referenced study is most likely due to the lower impact of Li substitution on the overall glass dissolution. In 13-93Li, K and Na are substituted with Li. K^+^ has a larger ionic radius (138 pm) than Li^+^ (76 pm), which diminishes the effect of substitution on overall molar volume, supported by the fact that the Si^4+^ release did not distinctively change with increasing Li substitution (Figure S1). Similarly, the Sr substitution seems to delay the formation of the CaP compared to unsubstituted glass (Figure S3). Delayed formation of the apatite phase has also been reported with SrO-containing silicophosphate glass in SBF, due to the slower formation rate of the Sr-substituted CaP compared to CaP without Sr.^27^ A similar effect has also been observed in Sr-containing S53P4 glass immersed in SBF.^28^

With the borosilicate glasses, the release of all ions was higher than 13-93. As previously explained, this is due to the faster dissolution of the borate phase in the glass: all ions leached from this phase before the silica phase. In addition, it has been shown that in borosilicate glasses, Ca^2+^ is preferentially connected to the borate phase over the silicate phase in the glass structure, which causes it to be released quickly in the solution.^29^ The higher bioactivity of the borosilicates was also seen as a quicker consumption of P^5+^ and Ca^2+^, associated with the CaP precipitation (Figure S3). The precipitates did not induce a decrease in the Ca^2+^ concentrations in the solution due to the higher Ca^2+^ release from the fast-dissolving borate phase compared to the lower release of Ca^2+^ from the silica phase in 13-93 (Figure S4).

Furthermore, with the borosilicates, the P^5+^ consumption occurs earlier than that in the silicate glasses. As the SBF was not refreshed, the lack of P^5+^ in the solution after 3 days prevented further Ca^2+^ consumption. The 13-93, 13-93Li, 13-93Sr, and 13-93B glasses showed similar release profiles, but the release of Si^4+^ and K^+^ was slower due to the decreased surface area compared to glasses with a smaller particle size (Figures S1 and S2). Furthermore, the CaP reactive layer precipitation seemed not to form with 13-93Li and 13-93Sr glasses, and the formation of the precipitate with 13-93B was delayed compared to that with B25 glass (Figures S3 and S4).

### 3.2 Cytocompatibility

#### 3.2.1 Human Adipose Stromal Cells and Human Lung Fibroblasts

The cytocompatibility of the Li-, Sr-, and B-substituted 13-93 BaGs was indirectly assessed by culturing cells in ionic glass extracts. Based on the cell culture results, the hASCs and WI-38 showed high viability in all the extracts (Figures 3 and 5). Similarly, 13-93 has shown good cytocompatibility with mesenchymal stem cells in previous studies,^30,31^ and according to our results, Li^+^- and Sr^2+^-containing extracts appeared beneficial for hASC proliferation. The number of hASCs in the 13-93Sr extract was significantly higher than that in the control at the 7-day time point and higher than those in the control and 13-93 extract after 14 days (Figure 4). Furthermore, the hASC number in the 13-93Li extract exceeded the control at all time points and the 13-93 extract after 7 and 14 days. In prior studies, Li^+^ has been demonstrated to accelerate the proliferation of mesenchymal and neural stem cells when the Li^+^ concentration is under 5 mM (35 ppm).^32,33^ These results are concordant with our results, where the Li^+^ levels did not exceed 25 ppm during the culture (Figure 18), even though we detected a slight increase in the ion concentrations in the medium during cell culturing, which might have been due to dissolution of smaller glass particles than the size range.

The WI-38 cells showed high viability, proliferation, and normal morphology when cultured with 13-93, 13-93Li, and 13-93Sr extracts (Figures 5, 6, and 15), as described previously.^21,34^ Shan et al.^34^ reported that Li disilicate glass ceramics showed high viability and normal morphology of gingival fibroblasts. Furthermore, Massera et al.^21^ showed that culturing human gingival fibroblasts on SrO-added S53P4 discs enhanced the cells’ growth and proliferation.

While the hASCs and WI-38s cultured in 13-93, 13-93Li, and 13-93Sr extracts exhibited well-organized and aligned cytoskeletons, the presence of B^3+^ in the extracts significantly decreased the number and increased the cell size of hASCs and WI-38 (Figures 4, 6, and 15). Larger hASCs with reduced proliferation cultured in borosilicate glass extracts were also reported earlier.^35^ Boron levels above 1 μg/mL (1 ppm, 0.09 mM) have been previously shown to decrease the cell viability and proliferation of hBMSC and MC3T3-E1 cells.^36,37^ In the present study, the viability of hASCs and WI-38 was high in 13-93B extracts, even though the ion concentration measurements from the cell culture mediums during culture revealed that the cells were exposed to boron levels of 48–82 ppm (4.4–7.6 mM).

Additionally, the B^3+^ levels may have an effect on cell behavior. In the study by Fu et al.,^38^ B^3+^ concentrations above 0.65 mM (26 ppm) in 13-93-based extract medium negatively affected bone marrow stromal cell proliferation. This concentration is significantly higher than 0.09 mM (3.6 ppm) reported for B^3+^ alone, suggesting that cells may tolerate higher B^3+^ concentrations as part of the ion mix from the BaG. To compare, the hASCs and WI-38 in the current study experienced B^3+^ concentrations of max. 79 and 62 ppm, respectively (Figure 18C). However, even though the borosilicate glasses inhibit the proliferation of cells in static conditions, they have not been reported to have such effects in dynamic conditions, for example, in vivo.^39^ Static conditions may enable the accumulation of ions and high local ion concentrations, whereas, in dynamic conditions, the ion concentrations stay below the toxic limits.^39^

In addition to the inhibitory effect on proliferation, the 13-93B extract distorted the morphology of hASCs and WI-38, having a more prominent effect on WI-38 (Figure 15). The hASC’s cytoskeletons were less organized, and the cells were larger, whereas WI-38 cultured in the 13-93B extract were significantly rounder and larger than the cells in the control media. Concurrent with the present study, rat BMSCs cultured on boron nitride nanotube-coated surfaces showed boron-associated widespread cells.^40^

However, the cells cultured in BaG extracts are exposed to a mixture of ions, and therefore, it is difficult to discuss the effects of a single ion type. As detected in the dissolution studies, replacing Si with B increases the glass reactivity (Figures S1–S4) and the pH of the buffer solution (Figure 1). For example, high Ca^2+^ levels (>10 mM, 400 ppm) have been associated with inhibited cell proliferation.^41^ In the present study, the ion concentration measurements from the cell culture media showed that the hASCs and WI-38 cultured in 13-93B extracts experienced 3- or 4-fold Ca^2+^ concentrations compared to the concentrations in their control medium (Figure 20A, B). The higher reactivity of 13-93B and higher concentrations of Ca^2+^ may explain an inhibitory effect on the proliferation of hASCs and WI-38.

#### 3.2.2 Human Urothelial Cells

The undiluted extracts did not support the viability or proliferation of the hUCs (Figures 7 and 8), and the cells adopted an abnormal elongated morphology at 3 days and, at 7 days, a distinctively changed morphology in all of the extracts. Diluting the extracts to 1:10 increased the hUC viability. However, distinctive morphological changes were seen in 13-93, 13-93Li, and 13-93B extracts (Figures 9 and 16). The cell morphology changed into a transparent, planar layer of large cells with nondistinct cell borders. However, a morphologically normal layer of hUCs was revealed under the planar layer (data not shown). In a previous study, a spinal cord injury provoked a similar change in the morphology of rat urothelium, which might have been due to stress hormones.^42^ Therefore, one option is that the distinctive morphological change may be due to chemical stress caused by the BaG extracts. The fact that less change was seen for the 1:100 diluted extracts supports this theory; however, more research is needed to confirm the nature and cause of the morphological changes observed.

One explanation for the cytotoxic effect of the undiluted BaG extracts on hUCs might be the pH increase induced by the dissolution of the glasses (Figure 1). hUCs are used for acidic pH, as the pH of the urine is typically between 4.5 and 8, of which pH over 7 is considered alkaline urine.^43^ Diluting the extracts decreased the ion concentrations and was also assumed to induce a more minor increase in pH than the undiluted extracts, possibly explaining the higher hUC viability in the diluted extracts (Figures 9 and 10). Previously, cytotoxic effects on human epithelial cells and human epithelial-like lung carcinoma cells have been reported, and they were associated with the pH increase by BaG dissolution.^44,45^ In addition, the cytotoxic effect was not detected with lower concentrations of BaGs. Furthermore, Raja et al.^46^ evaluated extracts from up to 10% Zn-substituted phosphate-based glass with human bladder urothelial cells and found that the extracts supported the viability and proliferation of the cells. However, they extracted glass discs instead of glass particles, and due to the lower surface area, the ion concentrations were expected to be lower in the study by Raja et al. They also report only a negligible change in the cell culture medium’s pH after the glasses’ extraction, which might also explain the excellent tolerance of the extracts by the urothelial cells.^46^ In the current study, the higher glass surface area during extraction probably led to a higher pH of the extract. However, we used different cell types and glass compositions; thus, no direct comparisons between these studies can be made. Furthermore, cell types have different resistances to each ion or mixture of ions. Further, the immortalized cell lines might be more resistant than nonimmortal cell lines.

Interestingly, the 1:10 diluted 13-93Sr extract induced relatively minor changes in hUC morphology compared to the other 1:10 diluted extracts (Figures 9 and 16), even though, in the dissolution study, the 13-93Sr glass induced a similar pH increase compared to the other silicate glasses (Figure 1). Therefore, another explanation for the poor hUC response of 13-93, 13-93Li, and 13-93B extracts might be the high Ca^2+^ levels (up to 177 ppm, Figure 21A) in the extracts. The Ca^2+^ level in the undiluted 13-93Sr extract was lower (18 ppm) than in the other glasses because Ca–Sr substitution decreased the amount of Ca in 13-93Sr. The 1:10 diluted 13-93, 13-93Li and 13-93B extracts had similar Ca^2+^ levels to the undiluted 13-93Sr extract (13–19 ppm, Figure 21B). Further, the 1:100 diluted extracts had 3–4 ppm of Ca^2^ (Figure 21C), close to the Ca^2+^ concentration in the hUC culture medium (4.3 ppm, Table 4).

In the diluted extracts, the viability of the hUCs increased, and their morphology was normalized. However, abnormal morphology was still noted in 1:10 diluted 13-93, 13-93Li, and 13-93B extracts (Figure 9 and 16). Accordingly, the threshold ion concentration for inducing significant morphological changes lies between the 1:100 and 1:10 dilutions. Previously, Ca^2+^ levels up to 100 ppm have shown no toxic effects on epithelial cells.^47^ In another study, when the extracellular Ca^2+^ levels were increased from 0.09 mM (3.6 ppm) to 4.0 mM (160 ppm), the growth rate of the urothelial cells was significantly reduced.^48^ Within 48 h, the cells formed stratified epithelium. However, we speculated that the decreased viability of the hUCs in the present study could not be explained solely by extracellular Ca^2+^ levels but rather by a combination of effects of the ion mixtures from the BaGs and their effects on the pH of the cell culture medium.

#### 3.2.3 Human Umbilical Vein Endothelial Cells

The HUVECs remained viable and proliferated well in all the extracts (Figures 11–14), which supports the angiogenic potential of the Li-, Sr-, and B-substituted BaGs published in previous studies.^4,49^ Previously, Li^+^ concentrations up to 15 ppm, similar to the undiluted 13-93Li extract in the present study (Figure 18A), have been reported to promote HUVEC proliferation in Li-BaG extracts compared to the BaG extract and medium alone.^4,49^ Based on our results, HUVECs tolerate higher ion concentrations than hUCs and higher B^3+^ concentrations than hASCs and WI-38, suggesting ion resistance differs between cell types. Bromet et al.^200^ cultured HUVECs in direct contact with borate BaG particles and noticed a decrease in HUVEC proliferation at 2.5 mg/mL borate glass concentrations. They did not measure the ion concentrations in the medium, but reported that the same glass released 480 ppm B^3+^ ions in 24 h in SBF, much more than in the undiluted EGM extracts in the current study (79 ppm, Figure 18C).

Diluting the 13-93Li, 13-93Sr, and 13-93B extracts to 1:10 and 1:100 positively affected HUVEC proliferation (Figure 14). The ion concentrations of diluted extracts are very similar, except for the small concentrations of Sr^2+^ and B^3+^ in the diluted extracts (max. 33 and 8 ppm, respectively, Figure 19). Previously, the beneficial effects of Sr^2+^ and B^3+^ on HUVECs have been reported, but they have had lower ion concentrations than our results.^8,50^ Sr-BaG extracts with Sr^2+^ concentrations of 6.3 ppm enhanced the angiogenesis of HUVECs similar to BaG extracts without Sr^2+^ but significantly better than the medium alone.^8^ In addition, B^3+^ concentrations of 55 ± 5 μM (0.6 ppm) from 45S5–B BaGs increased the HUVEC proliferation, migration, and formation of tubules.^50^ Furthermore, the Ca^2+^ levels experienced by HUVECs during the culture were higher than for all the other cell types (Figure 22), suggesting that HUVECs are less sensitive to Ca^2+^ levels than hASCs, WI-38, or hUCs.

Low Li^+^, Sr^2+^, and B^3+^ concentrations benefit HUVECs, but there are no notable differences between the ions. Therefore, the beneficial effects of the BaG dissolution products might be more due to the ion combinations from the BaGs rather than Li^+^, Sr^2+^, or B^3+^ alone. Si^4+^ released from silica-based ceramic materials has been shown to increase the proliferation of endothelial cells in vitro and angiogenesis in vivo.^51,52^ In addition, B^3+^ and Si^4+^ ions have been suggested to have a synergetic effect on angiogenesis.^53^ Furthermore, the HUVEC morphology was typical in the undiluted and diluted extracts (Figure 17). However, it should be noted that the actin filaments had organized in the cell edges in the HUVECs cultured in undiluted 13-93Sr and 13-93B extracts and 1:10 diluted 13-93 extract (Figure 17).

## 4 Conclusions

The undiluted 13-93, 13-93Li, and 13-93Sr extracts were beneficial for the viability and proliferation of hASCs, WI-38, and HUVECs. The presence of B^3+^ resulted in decreased viability and morphological changes in hASCs and WI-38. The hUCs did not remain viable in the undiluted BaG extracts, probably due to too alkaline pH or high ion concentrations, especially Ca^2+^. Diluting the extracts to 1:10 dilution induced a distinct morphology change in hUCs; however, diluting the extracts to 1:100 dilution normalized the hUC morphology. The 13-93, 13-93Li, 13-93Sr, and 13-93B BaGs seem beneficial for soft tissue engineering but in a dose-dependent manner.

## 5 Materials and Methods

### 5.1 Manufacturing of the Bioactive Glass Granules

Bioactive glass 13-93 and three series of 13-93-based Li-, Sr-, or B-substituted bioactive glasses were prepared from analytical grade K_2_CO_3_, Na_2_CO_3_, (NH_4_)H_2_PO_4_, (CaHPO_4_)(2(H_2_O)), CaCO_3_, MgO, Li_2_CO_3_, SrCO_3_, H_3_BO_3_, and Belgian quartz sand. The glass compositions are presented in Table 1. One hundred gram batches of 13-93 and Sr*X* (where *X* = 5, 10, 15, or 20 mol %) were melted for 3 h at 1425 °C in a platinum crucible. The Li-substituted glasses, denoted as Li*X* (where *X* = 3, 6, 9, or 12 mol %), were melted for 3 h at 1400 °C. The borosilicate glasses B10, B15, B20, and B25 were melted for 3 h at 1400, 1325, 1275, and 1225 °C, respectively. The molten glasses were cast, annealed, crushed, and finally sieved into 500–1000 and 125–250 μm particles. The glass particles were dried at 120 °C for 1 h before use.

**Table 1:** Bioactive Glass Compositions Given in Mole Percentages

glass composition	**SiO**_**2**_	**CaO**	**Na**_**2**_**O**	**K**_**2**_**O**	**MgO**	**P**_**2**_**O**_**5**_	**Li**_**2**_**O**	**SrO**	**B**_**2**_**O**_**3**_
13-93	54.6	22.1	6.0	7.9	7.7	1.7			
Li3	54.6	22.1	4.5	6.4	7.7	1.7	3.0		
Li6	54.6	22.1	3.0	4.9	7.7	1.7	6.0		
Li9	54.6	22.1	1.5	3.4	7.7	1.7	9.0		
Li12 (13-93Li)	54.6	22.1		1.9	7.7	1.7	12.0		
Sr5	54.6	17.1	6.0	7.9	7.7	1.7		5.0	
Sr10	54.6	12.1	6.0	7.9	7.7	1.7		10.0	
Sr15	54.6	7.1	6.0	7.9	7.7	1.7		15.0	
Sr20 (13-93Sr)	54.6	2.1	6.0	7.9	7.7	1.7		20.0	
B10	49.2	22.1	6.0	7.9	7.7	1.7			5.5
B15	46.4	22.1	6.0	7.9	7.7	1.7			8.2
B20	43.7	22.1	6.0	7.9	7.7	1.7			10.9
B25 (13-93B)	41.0	22.1	6.0	7.9	7.7	1.7			13.7

### 5.2 Glass Dissolution

The crushed BaGs, sieved into 125–250 and 500–1000 μm (for the highest substitution levels only) particles, were used for the dissolution studies. Glass particles (75 mg) were immersed in 50 mL of simulated body fluid (SBF) and placed into a shaking incubator for 1, 2, 3, 7, and 14 days at +37 °C following the protocol proposed by the technical committee on Glasses for Medicine and Biotechnology (ICG-TC04).^54^ The SBF was prepared following the protocol published by Kokubo et al.^55^ The pH of the SBF was measured with a Mettler Toledo SevenMultimeter (accuracy ±0.02) before dissolution and at each time point. At each time point, the glass granules were filtered from the buffer solution, washed with acetone, and dried in a fume hood for 4 h and at +37 °C overnight. One milliliter of the buffer solution was recovered for ICP analysis and diluted with 9 mL of 1 M HNO_3_. The elements and wavelengths measured with inductively coupled plasma (ICP-OES) were silicon (Si, λ = 250.690 nm), calcium (Ca, λ = 317.933 nm), potassium (K, λ = 766.491 nm), phosphorus (P, λ = 253.561 nm), lithium (Li, λ = 610.365 nm), boron (B, λ = 249.678 nm), and strontium (Sr, λ = 460.733 nm). The pH and ion concentrations were measured for three parallel samples, and the results are presented as means with standard deviation or the error of measurement, depending on which one was higher.

### 5.3 Preparation of the Bioactive Glass Extracts

The BaG granules (13-93, 13-93Li, 13-93Sr, and 13-93B; Table 1) with a particle size of 500–1000 μm were used to prepare the extracts. The glass particles were sterilized twice with 70% Aa ethanol (Altia Oyj, Helsinki, Finland). Afterward, the particles were left to dry for 2 h at room temperature in a laminar hood. BaG granules were added to extraction mediums at 87.5 mg/mL and incubated for 24 h at +37 °C on cell culture dishes (diameter 10 cm), according to ref (56). The extraction medium for WI-38 and hASCs contained Dulbecco’s Modified Eagle Medium/Ham’s Nutrient Mixture F-12 (DMEM/F-12 1:1; Thermo Fisher Scientific, Waltham, MA, USA), supplemented with 1% antibiotics (100 U/ml penicillin and 0.1 mg/mL streptomycin; Lonza, BioWhittaker, Verviers, Belgium) and 1% l-glutamine (GlutaMAX I; Thermo Fisher). After incubation, the medium with BaG extracts for WI-38 and hASCs was sterile filtered with a 0.2 μm filter and supplemented with 5% human serum (HS; Biowest, Nuaillé, France).

For hUCs, the BaGs were extracted at 87.5 mg/mL in a medium containing EpiLife (Thermo Fisher) and 0.35% antibiotics. After incubation, the medium was supplemented with 1% EDGS and 0.1% 0.06 M CaCl_2_ (both from Thermo Fisher). The BaG extracts for the HUVECs were made in EBM-2 Endothelial Cell Growth Basal Medium (Lonza) at a concentration of 87.5 mg/mL of BaG particles and supplemented after sterile filtering with SingleQuots supplements, including fetal bovine serum (FBS), hydrocortisone, human FGF-β, VEGF, insulin-like growth factor (IGF), ascorbic acid, human epidermal growth factor (hEGF), GA-1000, and heparin (Lonza).

A new batch of BaG extracts was made for each 2-week experiment, so the maximum storage time for each extract was 14 days at +4 °C. No visible precipitate was formed during this time. The BaG extracts were used as the cell culture medium in the cell cultures.

### 5.4 Determination of Ion Concentrations of the Bioactive Glass Extracts

The ion concentrations of the control mediums and extracts were measured with ICP-OES (Agilent Technologies, Santa Clara, CA, USA) during the cell culture at each medium change (0, 3, 5, 7, 10, 12, and 14 days). The samples were centrifuged to remove the possible precipitates, and 1 mL of the supernatant was collected and diluted in 9 mL of distilled water and spiked with 50 μL of HNO_3_. The levels of Si^4+^, Ca^2+^, K^+^, P^5+^, Li^+^, Sr^2+^, and B^3+^ were measured from the cell culture mediums before culture similarly to the dissolution part, but the levels of magnesium (Mg^2+^, λ = 279.553 nm) were also measured. During the cell culture, the concentrations of Li^+^, Sr^2+^, B^3+^, and Ca^2+^ were measured from the extracts.

### 5.5 Cell Sourcing and Isolation

#### 5.5.1 Human Adipose Stromal Cells

The hASCs were isolated in our laboratory from adipose tissue samples as previously described,^57^ with the approval of the Ethics Committee of Pirkanmaa Hospital District (R15161) and with written informed consent from the patient. Shortly, the adipose tissue samples were digested with collagenase type I (1.5 mg/mL, Thermo Fisher), after which they were centrifuged and filtered. The hASCs were expanded in T75 polystyrene flasks (Thermo Fisher) in DMEM/F-12 (Thermo Fisher) medium, supplemented with 5% HS (Biowest) and 1% L-glutamine (GlutaMAX, Thermo Fisher) and 1% antibiotics (100 U/ml penicillin and 0.1 mg/mL streptomycin, Lonza). This medium composition is later defined as the control medium (BM). The hASCs from three donors in passages 4–5 were used in the cell culture experiments.

#### 5.5.2 Human Lung Fibroblasts

The human lung fibroblasts used in this study were commercially available WI-38 fibroblasts (Culture Collection, Public Health England, United Kingdom). The WI-38 were expanded in BM, and the experiments were performed in passage 25.

#### 5.5.3 Human Urothelial Cells

The hUCs were isolated as previously described.^58,59^ The urothelium tissue samples were obtained from normal ureters from child donors during routine elective surgery at Tampere University Hospital, with the approval of the Ethics Committee of Pirkanmaa Hospital District (R07160) and written informed consent from the parents. Shortly, the tissue pieces were cut into smaller fragments and incubated overnight in a stripping solution containing 0.01% HEPES buffer (1 M, Sigma-Aldrich), 4 × 10^–3^% aprotinin (1 KIU/μL; Sigma-Aldrich), 0.1% EDTA (Sigma-Aldrich), and 0.01% penicillin/streptomycin (Lonza) in Hank’s balanced salt solution (HBSS, Thermo Fisher, without Ca^2+^ and Mg^2+^) to ease the removal of the urothelial layer. The following day, the urothelial sheets were separated from the stroma and digested in 0.1% trypsin (Lonza) for 30 min at 37 °C in a shaking water bath. The 10% HS (Biowest) in HBSS was used to inactivate the trypsin, and the resulting suspension was centrifuged and resuspended in EpiLife medium (Thermo Fisher) supplemented with 1% of EpiLife defined growth supplement (EDGS; Thermo Fisher), 0.1% of CaCl_2_ (Thermo Fisher), and 0.35% of antibiotics (100 U/μL penicillin and 0.1 mg/μL streptomycin, Lonza). The hUCs were expanded in CellBIND culture flasks (Corning, MA, USA). The cells from three different donors in passages 4–7 were used in the experiments, and the supplemented EpiLife medium (EPI) was considered the control medium for the hUCs.

#### 5.5.4 Human Umbilical Vein Endothelial Cells

The HUVECs were isolated from human umbilical cord veins as described previously.^60,61^ The human umbilical cords were obtained from scheduled cesarean sections at Tampere University Hospital (permission R13019 from the Ethics Committee of the Pirkanmaa Hospital District, Tampere, Finland) with written informed consent from the parents. In brief, the umbilical cord was separated from the placenta, and the umbilical vein was cannulated with a 20G needle. The vein was perfused with PBS to wash out blood and infused with 0.05% collagenase I. The cord was incubated in a water bath at 37 °C for 15 min, after which the collagenase solution containing HUVECs was rinsed from the cord with PBS. The cells were centrifuged, washed with medium, centrifuged again, and finally resuspended in EGM-2 Endothelial Cell Growth Medium (Lonza), where the cells were cultured and expanded. This medium is also denoted as the control medium for HUVECs (EGM). The HUVECs from three donors in passage 6 were used in the cell culture experiments.

### 5.6 Cell Seeding

The hASCs, WI-38, and HUVECs were plated on a 24-well plate (Nunc, Roskilde, Denmark) in 1 mL of control medium with a 1000 cells/cm^2^ density. The hUCs were plated on CellBind 24-well plates (Corning) at 10,000 cells/cm^2^ density. The cells were allowed to adhere for 24 h in control mediums, after which the mediums were replaced with BaG extracts or fresh control medium. Altogether, there were 31 different culturing conditions (Table 2): BM, EPI, or EGM as a control, and four different BaG extracts (13-93 and Li-, Sr-, and B-substituted 13-93) in each medium. Additionally, 1:10 and 1:100 extract dilutions in EPI and EGM were studied for hUCs and HUVECs. The initial ion concentrations of the mediums and extracts are presented in Tables 3, 4, and 5. The cells were cultured at 37 °C in a humidified atmosphere with 5% CO_2_, and the mediums were changed three times a week.

**Table 2:** Medium Compositions Used in the Study

**medium**	**undiluted**	**diluted**1:10	**diluted**1:100
BM	x		
13-93 in BM	x		
13-93Li in BM	x		
13-93Sr in BM	x		
13-93B in BM	x		
EPI	x		
13-93 in EPI	x	x	x
13-93Li in EPI	x	x	x
13-93Sr in EPI	x	x	x
13-93B in EPI	x	x	x
EGM	x		
13-93 in EGM	x	x	x
13-93Li in EGM	x	x	x
13-93Sr in EGM	x	x	x
13-93B in EGM	x	x	x

**Table 3:** Initial Ion Concentrations of the BM-Based Extracts in the Study

	**Ca**^**2+**^	**K**^**+**^	**Mg**^**2+**^	**P**^**5+**^	**Si**^4+^	**Li**^**+**^	**Sr**^**2+**^	**B**^**3+**^
**culture medium**	**(ppm)**	**(ppm)**	**(ppm)**	**(ppm)**	**(ppm)**	**(ppm)**	**(ppm)**	**(ppm)**
BM	40.3	153.2	16.4	30.8				
BM 13-93	85.7	208.9	30.8	18.1	45.1			
BM 13-93Li	120.5	183.5	39.8	30.0	60.4	18.6		
BM 13-93Sr	37.1	263.9	38.9	26.3	59.5		221.0	
BM 13-93B	128.0	279.5	43.9	24.2	63.0			49.9

**Table 4:** Initial Ion Concentrations of the EPI-Based Extracts in the Study

	**Ca**^**2+**^	**K**^**+**^	**Mg**^**2+**^	**P**^**5+**^	**Si**^4+^	**Li**^**+**^	**Sr**^**2+**^	**B**^**3+**^
**culture medium**	**(ppm)**	**(ppm)**	**(ppm)**	**(ppm)**	**(ppm)**	**(ppm)**	**(ppm)**	**(ppm)**
EPI	4.3	116.3	14.6	45.7				
EPI 13-93	125.5	245.9	48.3	30.6	59.3			
EPI 13-93Li	106.7	138.8	39.9	47.8	58.8	20.9		
EPI 13-93Sr	16.6	255.8	44.7	43.4	59.7		318.5	
EPI 13-93B	144.9	309.3	56.3	25.6	69.5			76.7
EPI 13-93 1:10	15.2	124.3	17.1	43.6	4.7			
EPI 13-93Li 1:10	13.5	113.4	16.2	44.4	4.7			
EPI 13-93Sr 1:10	3.5	120.4	15.9	42.2	4.2		26.7	
EPI 13-93B 1:10	15.0	123.8	16.7	41.5	4.9			6.6
EPI 13-93 1:100	3.6	111.1	13.6	43.0				
EPI 13-93Li 1:100	3.5	109.4	13.6	43.3				
EPI 13-93Sr 1:100	2.5	110.0	13.5	42.6			2.6	
EPI 13-93B 1:100	3.8	109.8	13.8	42.8				0.7

**Table 5:** Initial Ion Concentrations of the EGM-Based Extracts in the Study

	**Ca**^**2+**^	**K**^**+**^	**Mg**^**2+**^	**P**^**5+**^	**Si**^4+^	**Li**^**+**^	**Sr**^**2+**^	**B**^**3+**^
**culture medium**	**(ppm)**	**(ppm)**	**(ppm)**	**(ppm)**	**(ppm)**	**(ppm)**	**(ppm)**	**(ppm)**
EGM	57.9	144.6	205.4	21.3				
EGM 13-93	173.3	253.4	226.8	15.1	61.3			
EGM 13-93Li	141.4	159.3	216.0	18.4	60.1	11.5		
EGM 13-93Sr	53.5	260.6	216.1	15.1	58.6		245.3	
EGM 13-93B	192.2	296.4	226.4	11.5	63.0			65.9
EGM 13-93 1:10	73.5	164.0	213.0	21.6	5.0			
EGM 13-93Li 1:10	72.4	156.3	214.6	21.2	5.3			
EGM 13-93Sr 1:10	61.3	167.0	214.2	21.2	5.3		27.9	
EGM 13-93B 1:10	77.4	172.8	214.4	19.0	6.4			7.2
EGM 13-93 1:100	62.6	153.5	212.1	18.3				
EGM 13-93Li 1:100	61.6	151.8	208.8	17.5				
EGM 13-93Sr 1:100	62.0	156.2	217.7	20.8			2.9	
EGM 13-93B 1:100	62.2	153.1	211.8	19.7				0.7

### 5.7 Cell Viability

The viability of the cells was qualitatively assessed after 3, 7, and 14 days of cell culturing using live/dead fluorescence staining (L/D kit, Thermo Fisher). Briefly, the cells were incubated for 45 min in a working solution containing 0.25 μM EthD-1 (stains dead cells red) and 0.5 μM Calcein-AM (stains living cells green). The cells were imaged with a fluorescence microscope (Olympus IX51, Tokyo, Japan) immediately after the incubation.

### 5.8 Cell Proliferation

The cell proliferation at 3, 7, and 14 days was assessed by measuring the DNA amounts using a CyQUANT Cell Proliferation Assay kit (Thermo Fisher) according to the manufacturer’s protocol. In brief, the cells were lysed with 0.1% Triton-X 100 buffer (Sigma-Aldrich) and stored at −70 °C until analysis. Three parallel 20 μL samples of each well were pipetted to a 96-well plate and mixed with 180 μL of a working solution containing a CyQUANT GR dye and cell lysis buffer. The 480/520 nm fluorescence was measured with a multilabel counter (Victor 1420; Wallac, Turku, Finland).

### 5.9 Immunocytochemical Staining

The morphologies of hASCs, WI-38, and HUVECs, and hUC cytokeratin staining were evaluated with immunocytochemical staining after 14 days of cell culture. Shortly, the cells were fixed for 15 min at room temperature with 4% paraformaldehyde supplemented with 0.2% Triton-X 100. The hASCs, WI-38, and HUVECs were blocked overnight with 1% bovine serum albumin. The day after, they were stained with a 1:500 phalloidin-TRITC solution (Sigma-Aldrich) for 45 min at RT to evaluate the actin cytoskeleton organization. After fixing, the hUCs were incubated in primary antibody dilutions (1:250 AE1/AE3 pancytokeratin, Cytokeratin Pan Ab, Thermo Fisher) overnight at +4 °C. The following day, the cells were incubated in the secondary antibody solution (1:800 Alexa Fluor 488, Thermo Fisher) diluted in 1% BSA for 45 min at +4 °C. Finally, the nuclei of all of the cell types were stained with DAPI (1:2000, blue fluorescence, Sigma-Aldrich). The cells were imaged immediately after staining with a fluorescent microscope (Olympus).

### 5.10 Statistical Analyses

The statistical analyses of the CyQUANT data were performed with SPSS Statistics 25 (IBM, Armonk, NY, USA). The CyQUANT analysis was repeated with three cell lines and three parallel samples (*n* = 9) with hASCs and WI-38. In the case of hUCs and HUVECs, the CyQUANT was performed only for one cell line (*n* = 3). The data were statistically analyzed with a nonparametric Kruskal–Wallis test. The Bonferroni correction based on the number of planned comparisons was used to minimize the error produced by familywise calculations. The results were considered statistically significant when the adjusted *p*-value was <0.05 and highly significant when the *p*-value was <0.001. All pH data are presented as means accompanied by the error of measurement (0.05), and the ICP data as means with the measurement error (5% of the measured concentration) or standard deviation, whichever was higher.

## Supporting Information Available

Figure S1. Release of silicon from the glasses during the 14-day dissolution in SBF; Figure S2. Potassium concentration of the SBF during the 14-day dissolution of the glasses; Figure S3. Phosphorus concentration of the SBF during the 14-day dissolution of the glasses; Figure S4. Calcium concentration of the SBF during the 14-day dissolution of the glasses (PDF)
