# Supplementary material for: Ionic Dissolution Products of Lithium-, Strontium-, and Boron-Substituted Silicate Glasses Influence the Viability and Proliferation of Adipose Stromal Cells, Fibroblasts, Urothelial and Endothelial Cells
Source: ACS Omega. 2024 Dec 4;9(50):49348–67. doi: 10.1021/acsomega.4c06587 (PMC11656255; doi:10.1021/acsomega.4c06587)
Supplement: Supplementary file 1 — ao4c06587_si_001.pdf [file ao4c06587_si_001.pdf]

Supporting Information

Ionic dissolution products of lithium-, strontium- and boron-substituted silicate glasses influence the viability and proliferation of adipose stromal cells, fibroblasts, urothelial and endothelial cells

Inari Lyyra[*,1], Mari Isomäki[1], Heini Huhtala[2], Minna Kellomäki[1], Susanna Miettinen[3,4], Jonathan Massera[1,¶], Reetta Sartoneva[3,4,5,¶]

[1] Faculty of Medicine and Health Technology, Tampere University, Korkeakoulunkatu 3, Tampere FI-33720, Finland

[2] Faculty of Social Sciences, Tampere University, Arvo Ylpön katu 34, Tampere FI-33520, Finland

[3] Faculty of Medicine and Health Technology, Tampere University, Arvo Ylpön katu 34, Tampere FI-33520, Finland

[4] Research and Development and Innovation, Tampere University Hospital, Wellbeing Services County of Pirkanmaa, Arvo Ylpön katu 6, Tampere FI-33521, Finland

[5] Department of Obstetrics and Gynaecology, Seinäjoki Central Hospital, South Ostrobothnia Wellbeing Services County, Hanneksenrinne 7, Seinäjoki FI-60220, Finland

[*]Corresponding author

[¶] These authors contributed equally to this work.

Pages S1-S5

Contents

Figure S1. The release of silicon from the bioactive glasses during the 14-day dissolution in SBF

Figure S2. The potassium concentration of SBF with bioactive glasses during the 14-day dissolution

Figure S3. The phosphorus concentration of SBF with bioactive glasses during the 14-day dissolution

Figure S4. The calcium concentration of SBF with bioactive glasses during the 14-day dissolution

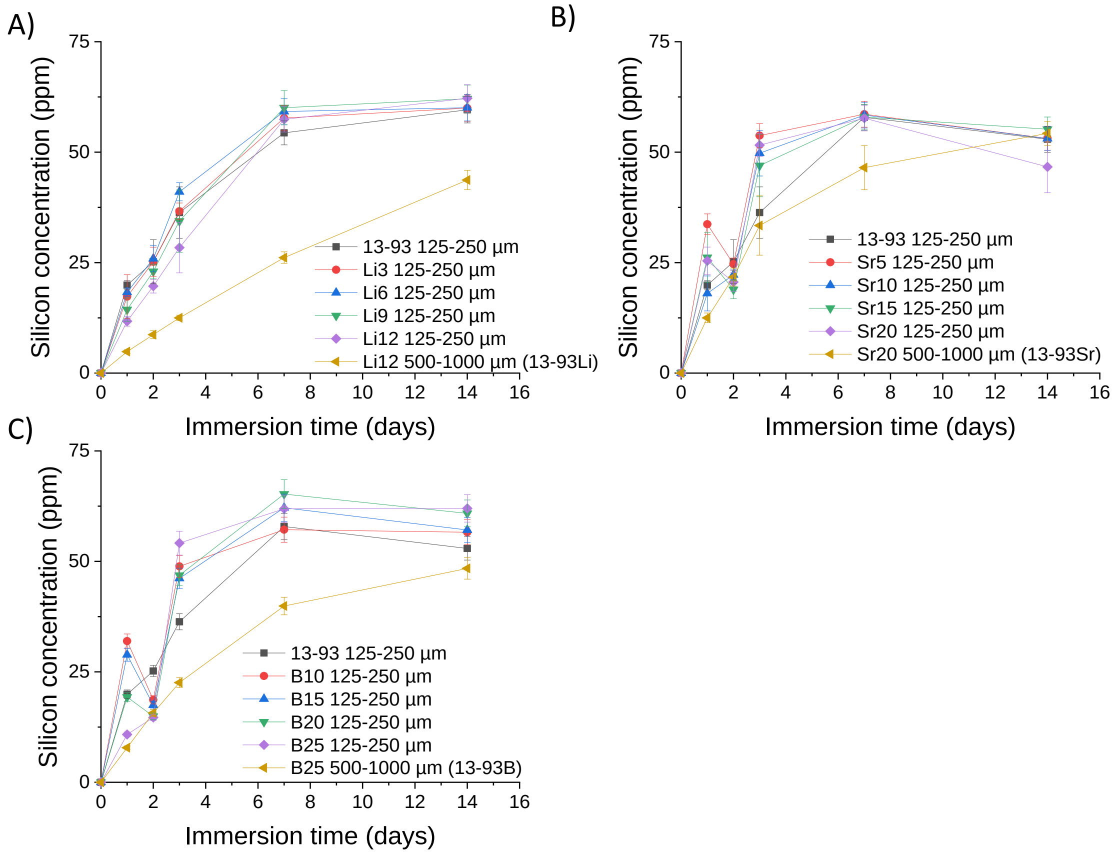

Figure S1. *The release of silicon from A) lithium (Li) -substituted, B) strontium (Sr) -substituted, and C) boron (B) -substituted 13-93 bioactive glasses during the 14-day dissolution in SBF. n = 3. The results are presented as means with a standard deviation or the measurement error.*

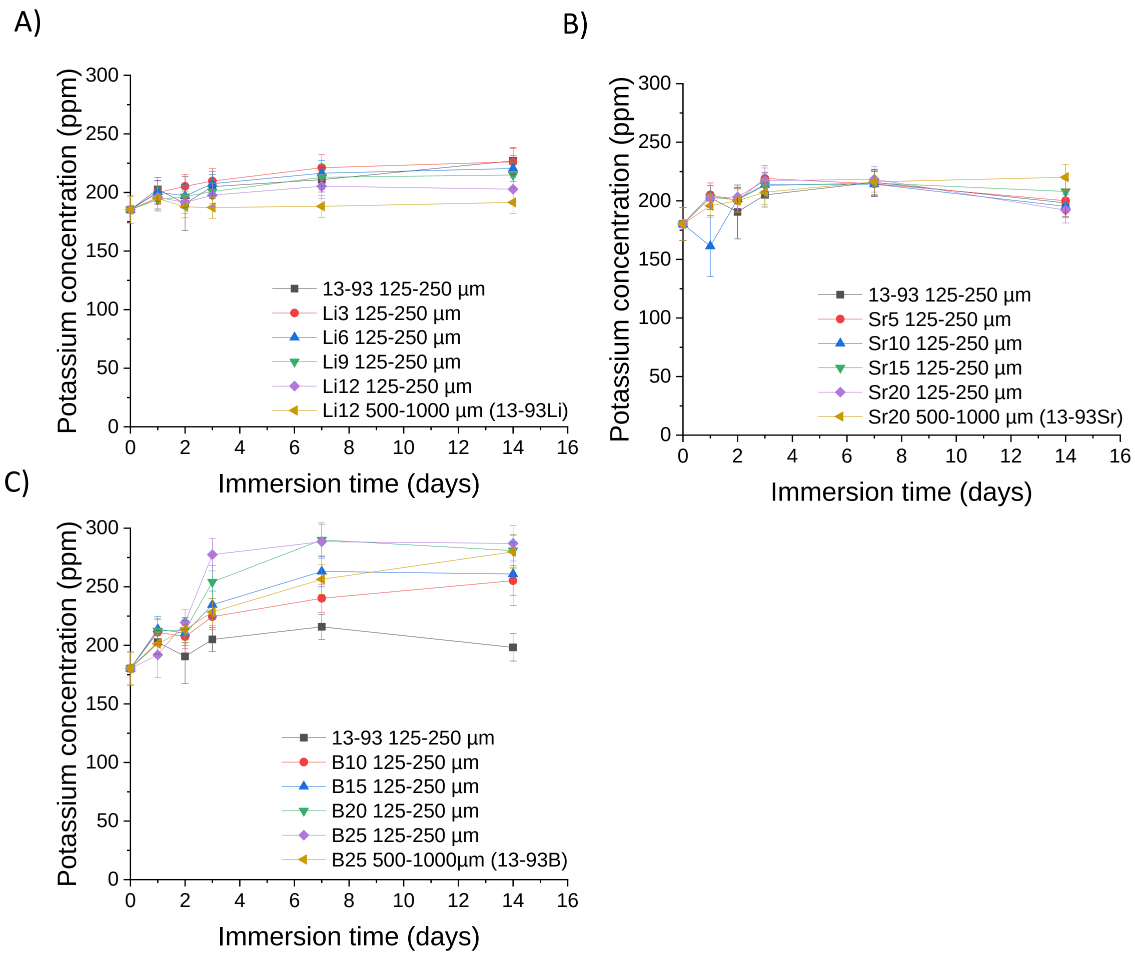

Figure S2. *The potassium concentration of SBF with A) lithium (Li) -substituted, B) strontium (Sr) -substituted, and C) boron (B) -substituted 13-93 bioactive glasses during the 14-day dissolution. n = 3. The results are presented as means with a standard deviation or the measurement error.*

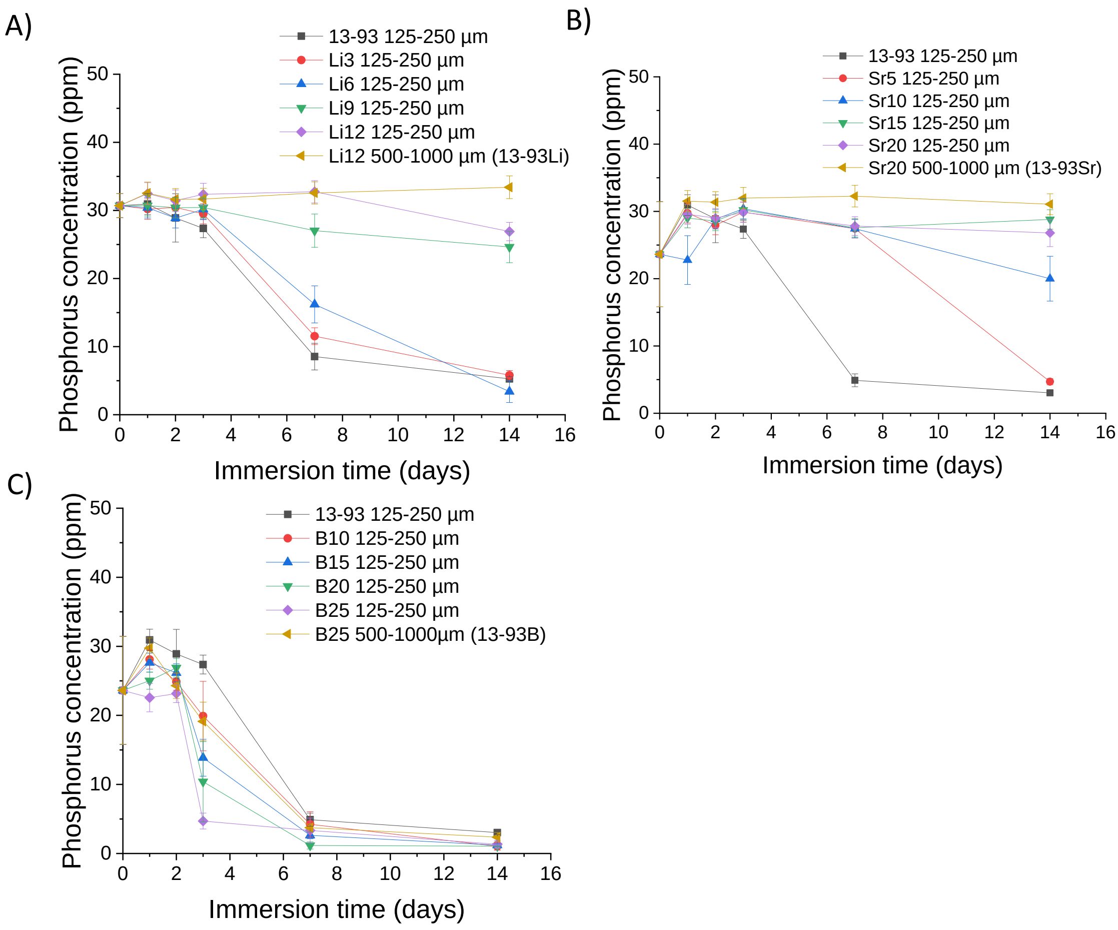

Figure S3. *The phosphorus concentration of SBF with A) lithium (Li) -substituted, B) strontium (Sr) -substituted, and C) boron (B) -substituted 13-93 bioactive glasses during the 14-day dissolution. n = 3. The results are presented as means with a standard deviation or the measurement error.*

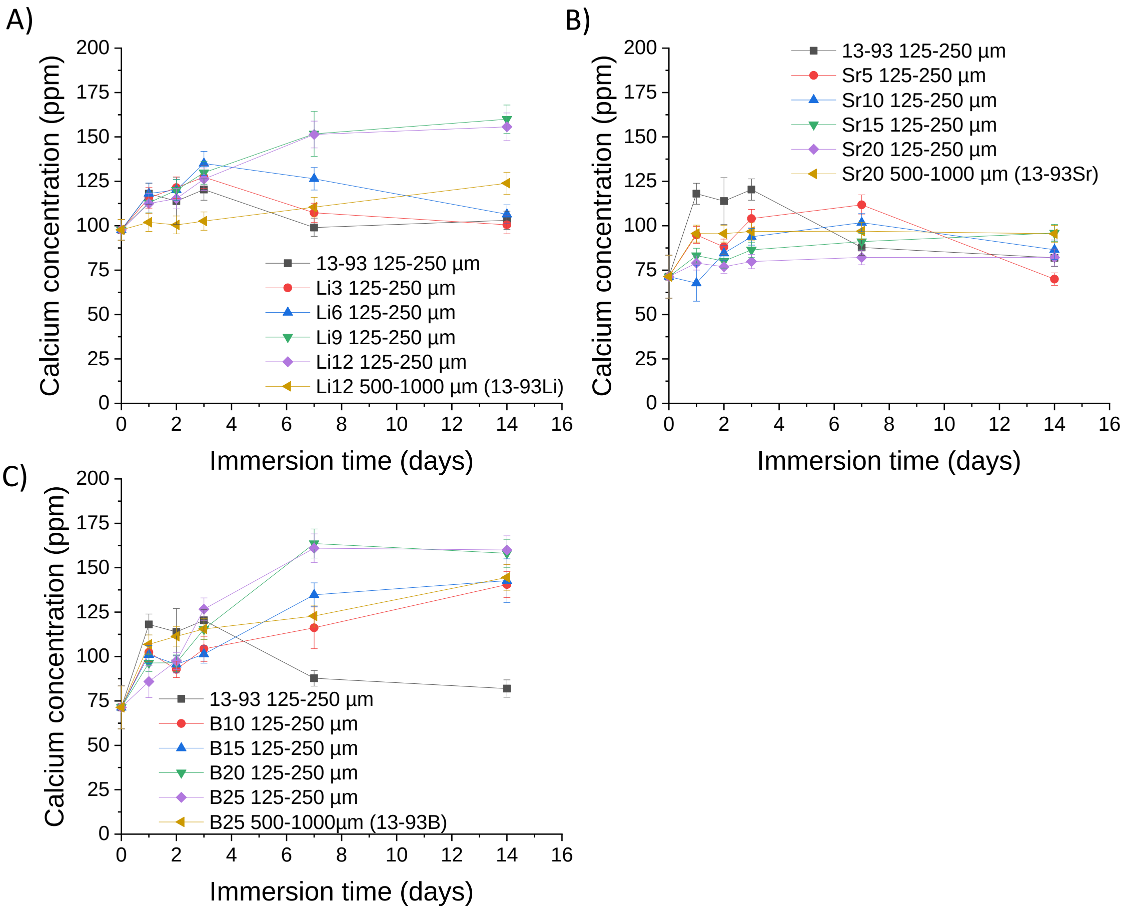

Figure S4. *The calcium concentration of SBF with A) lithium (Li) -substituted, B) strontium (Sr) -substituted, and C) boron (B) -substituted 13-93 bioactive glasses during the 14-day dissolution. n = 3. The results are presented as means with a standard deviation or the measurement error.*
